# A pathogen-specific isotope tracing approach reveals metabolic activities and fluxes of intracellular *Salmonella*

**DOI:** 10.1371/journal.pbio.3002198

**Published:** 2023-08-18

**Authors:** Karin Mitosch, Martin Beyß, Prasad Phapale, Bernhard Drotleff, Katharina Nöh, Theodore Alexandrov, Kiran R. Patil, Athanasios Typas

**Affiliations:** 1 Genome Biology Unit, European Molecular Biology Laboratory, Heidelberg, Germany; 2 Institute of Bio- and Geosciences, IBG-1: Biotechnology, Forschungszentrum Jülich GmbH, Jülich, Germany; 3 RWTH Aachen University, Computational Systems Biotechnology, Aachen, Germany; 4 Metabolomics Core Facility, European Molecular Biology Laboratory, Heidelberg, Germany; 5 Structural and Computational Biology Unit, European Molecular Biology Laboratory, Heidelberg, Germany; 6 Molecular Medicine Partnership Unit, European Molecular Biology Laboratory, Heidelberg, Germany; 7 BioInnovation Institute, Copenhagen, Denmark; 8 Medical Research Council Toxicology Unit, University of Cambridge, Cambridge, United Kingdom; UT Southwestern: The University of Texas Southwestern Medical Center, UNITED STATES

## Abstract

Pathogenic bacteria proliferating inside mammalian host cells need to rapidly adapt to the intracellular environment. How they achieve this and scavenge essential nutrients from the host has been an open question due to the difficulties in distinguishing between bacterial and host metabolites in situ. Here, we capitalized on the inability of mammalian cells to metabolize mannitol to develop a stable isotopic labeling approach to track *Salmonella enterica* metabolites during intracellular proliferation in host macrophage and epithelial cells. By measuring label incorporation into *Salmonella* metabolites with liquid chromatography–mass spectrometry (LC–MS), and combining it with metabolic modeling, we identify relevant carbon sources used by *Salmonella*, uncover routes of their metabolization, and quantify relative reaction rates in central carbon metabolism. Our results underline the importance of the Entner–Doudoroff pathway (EDP) and the phosphoenolpyruvate carboxylase for intracellularly proliferating *Salmonella*. More broadly, our metabolic labeling strategy opens novel avenues for understanding the metabolism of pathogens inside host cells.

## Abstract 2

How do intracellular pathogenic bacteria scavenge essential nutrients from the host? Difficulties in distinguishing between bacterial and host metabolites have made this challenging to address, but this study shows that 13C-mannitol allows direct tracking of the metabolism of Salmonella when growing inside host cells, revealing the food sources and metabolic pathways that intracellullar pathogens use.

## Introduction

Intracellular bacterial pathogens, like *Salmonella*, have evolved elaborate mechanisms to adapt, survive, and replicate within different niches inside host cells, which offer suboptimal growth conditions [1–3]. Entering into the host leads to massive changes in bacterial gene and protein expression [4,5], exemplified by the secretion of dozens of effector proteins into the host cytoplasm [6]. At the same time, bacteria must quickly reprogram their metabolism to adapt to the new environment and the metabolites it offers, restricts, or lacks.

Measuring metabolic activities of intracellular bacteria has been a longstanding challenge. In contrast to transcripts or proteins, which carry species identity within their sequence, most bacterial metabolites cannot be distinguished from those of the mammalian host, especially during intracellular infection when the two metabolic networks become closely intertwined. For *Salmonella* Typhimurium (*S*Tm), a facultative intracellular pathogen mainly replicating inside a *Salmonella-*containing vacuole (SCV), previous studies have approached this problem indirectly by inferring metabolic changes from transcriptomics [4] or proteomics [7] of isolated bacteria. Other studies have used gene knockout mutants to determine essential pathways for intracellular replication [7–9]. While such indirect approaches provide insights into the enzymatic pathways for intracellular growth, there is room for misinterpretation. For example, gene expression has been shown repeatedly to be a poor predictor of what cells need in a given condition [10,11]. Many metabolic enzymes and pathways are regulated at the level of activity rather than that of expression [12]. Moreover, gene mutants may abolish intracellular growth for other reasons beyond missing metabolic activity, such as the accumulation of toxic intermediates [13] or due to moonlighting functions of enzymes [14]. Thus, although powerful, genetic or gene expression approaches do not directly measure the metabolic activities of the pathogen.

To disentangle metabolic network activities and to quantitatively infer intracellular metabolic reaction rates (fluxes) in living cells, stable isotope labeling strategies combined with mass spectrometry (MS) or nuclear magnetic resonance (NMR) and metabolic modeling are the definitive methods [15,16]. However, separating intracellular metabolites in host–pathogen systems, and indeed in any compartmentalized systems such as eukaryotes [17,18], plants [19], or microbial communities [20], remains an analytical barrier, often turning out to be a showstopper for interpreting isotope labeling data. Several attempts have been made to work around this limitation: Labeling strategies have been designed and tailored to the specific question at hand. For example, Lewis and colleagues used dual ^13^C/deuterium tracing to resolve compartmentalized NADPH metabolism in mammalian cells [21], and Borah and colleagues combined multiple ^15^N labeling experiments to shed light on the nitrogen metabolism in intracellular *Mycobacterium tuberculosis* [22]. More generally, reporter proteins or peptides have been used in union with computational deconvolution to derive species-specific labeling patterns of amino acids [23–25]. More recently, stable isotope tracing in community-scale mass spectrometry analysis has been used to infer intra- as well as inter-species metabolite exchange [26].

Towards estimating metabolic activities in intracellular *Salmonella*, Götz and colleagues [27] supplied ^13^C-labeled glucose to infected epithelial colorectal adenocarcinoma cells (Caco-2) and determined labeling patterns of amino acids from isolated bacteria and host cells. Yet, glucose is utilized by both pathogen and host, leading to rapid “scrambling” of the labels across the two metabolic networks. Consequently, without additional experiments, such nonspecific labeling approaches cannot distinguish whether *S*Tm meets its demand for a specific amino acid by de novo production or through sequestration from the host. Xu and colleagues applied a reverse labeling approach, in which they pre-labeled *Salmonella* with deuterium to detect its dilution after infection in groups of metabolites by single-cell Raman spectroscopy. Thereby, they uncovered differences in intracellular lipid metabolism among *Salmonella* strains [28]. This approach is, however, limited to (i) metabolites that can be detected by Raman spectroscopy; and (ii) a short time-window as the growing cells will rapidly dilute the label.

To our knowledge, no bacterial-specific metabolic labeling approach has been described for intracellular infection before. Here, we present a stable-isotope tracing approach to specifically label and directly measure the metabolites of intracellularly growing *S*Tm. The approach is based on the inability of mammalian host cells—in contrast to *S*Tm—to metabolize the sugar alcohol mannitol. Using liquid chromatography–mass spectrometry (LC–MS), we quantified ^13^C-labeling patterns in various bacterial pathways during growth in two eukaryotic host cell lines. Tracing the breakdown of the ^13^C label throughout the metabolic pathways allowed us to identify metabolites that are de novo synthesized. Metabolic modeling and ^13^C metabolic flux analysis (^13^C MFA) were then used to unlock the identification of relevant carbon sources (besides mannitol) that enter *Salmonella*, delineate the routes of their metabolization, and estimate the major metabolic fluxes during infection, thereby providing hitherto unknown details of the intracellular *Salmonella* metabolism.

## Results

### Mannitol is metabolized only by intracellular *S*Tm, not the host

**Fig 1:**
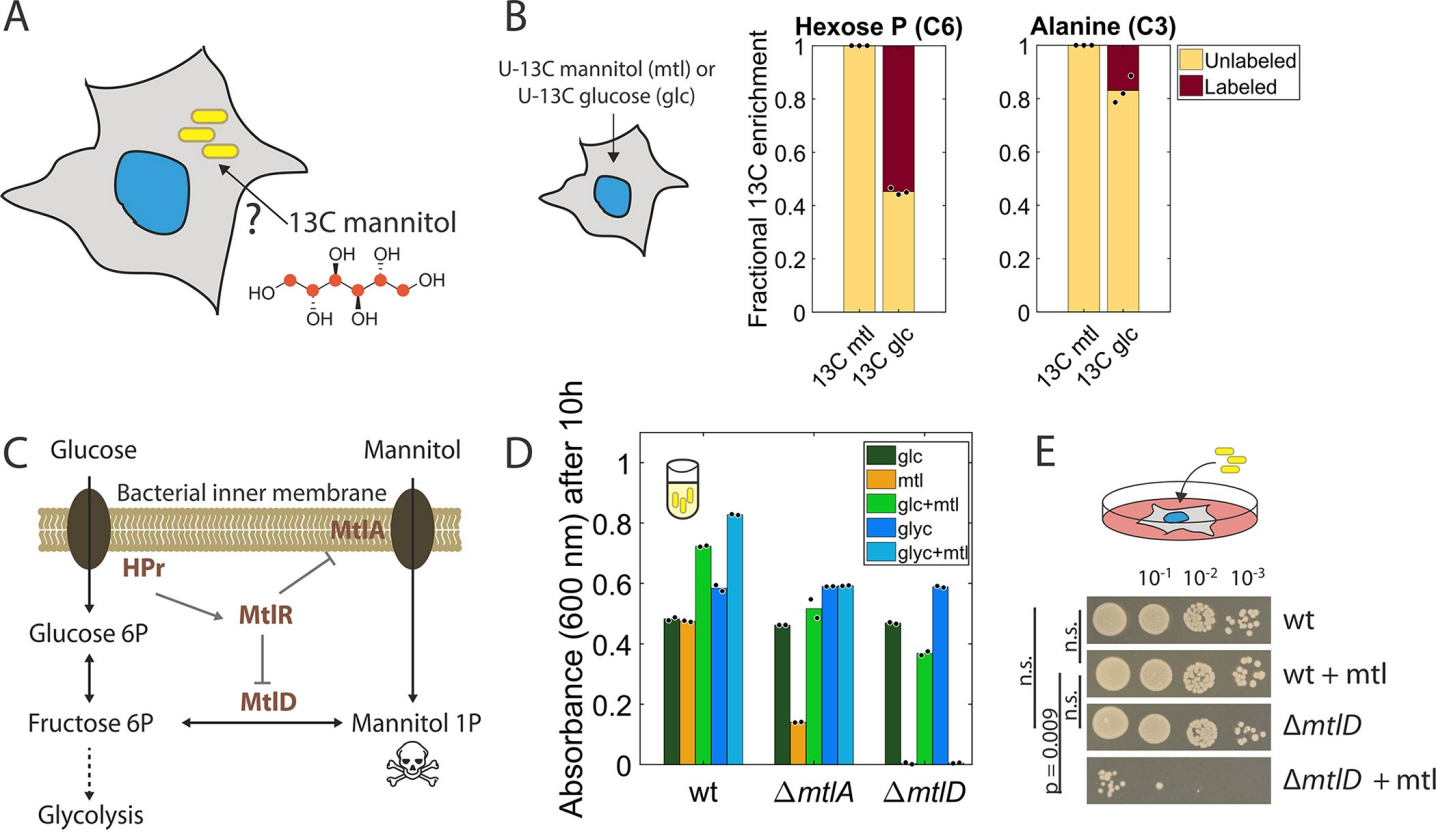
Mannitol is not metabolized by host cells, but internalized and used by intracellular *S*Tm. (A) Experimental concept: Can ^13^C-labeled mannitol, supplied to infected host cells, traverse across mammalian cell membranes without degradation and be taken up by intracellular *S*Tm (yellow)? (B) Fractional ^13^C enrichment of hexose-phosphates and alanine from RAW264.7 cells, determined 48 h after the addition of U-^13^C mannitol (mtl) or U-^13^C glucose (glc) into the glucose-containing cell culture medium (DMEM with 1 g/L glucose, Methods). ^13^C from labeled glucose is incorporated (labeled fraction present), but not from mannitol (no labeled fraction present). Graphs show averages from biological triplicates. (C) Mannitol metabolism in *S*Tm: Mannitol enters and is phosphorylated via MtlA; mannitol 1P (toxic when accumulating) is oxidized by MtlD to fructose 6P, where it enters glycolysis. The uptake and metabolization of mannitol are subject to glucose repression via the dephosphorylated phosphocarrier protein HPr, which enhances the activity of the repressor MtlR [32]. (D) Growth yield of *S*Tm wild type (wt), ∆*mtlA*, and ∆*mtlD*, in MOPS medium with amino acids (Methods), and with combinations of glucose (glc), glycerol (glyc), and mannitol (mtl), as the main carbon sources. Bars depict the averages of technical duplicates and data are representative of 2 independent experiments. (E) Intracellular *S*Tm wt and ∆*mtlD* isolated from RAW264.7 macrophages 20 hpi in a gentamicin protection assay (MOI = 100) supplemented +/− mannitol (mtl), and then serially diluted and spotted on an LB agar plate. The image is representative of 2 independent experiments in biological triplicates. The data underlying this figure can be found in S1 Data. glc, glucose; MOI, multiplicity of infection; *S*Tm, *Salmonella* Typhimurium.

To specifically label intracellular *S*Tm bacteria post-invasion of host cells, we searched for a carbon source that would (i) enter host cells, but not be utilized by them; (ii) be accessible and used by intracellularly growing bacteria; and (iii) minimally impair the native host and bacterial metabolism. *S*Tm can grow on a more diverse array of carbon sources [29] than mammalian cells [30,31]. Guided by the study by Steeb and colleagues [7] on nutrient availabilities for *S*Tm in a mouse infection model, we tested whether mannitol meets the above 3 requirements (Fig 1A).

To test the ability of host cells to metabolize mannitol, we supplemented two mammalian cell lines with either uniformly ^13^C-labeled mannitol (henceforth: U-^13^C mannitol) or, as a control, uniformly ^13^C-labeled glucose (henceforth: U-^13^C glucose) in their already glucose-containing cell culture medium (DMEM, Methods). We incubated the cells for 48 h to capture any incorporation of labeled mannitol during or after glucose consumption. The two cell lines, murine RAW264.7 macrophages, and human epithelial HeLa cells were chosen based on their biological relevance, and the distinct life cycles they enable for *S*Tm [33,34]. Isotope tracing using LC–MS for hexose-phosphates and alanine confirmed the expected incorporation of ^13^C-label from U-^13^C glucose, but no assimilation of U-^13^C mannitol (Figs 1B and S1A), consistent with past observations that mammalian cells are not able to metabolize mannitol [31].

*S*Tm, in contrast, has been known to efficiently metabolize mannitol, which is internalized and phosphorylated by the specific phosphotransferase (PTS) MtlA, and oxidized to fructose 6P by the mannitol 1-phosphate-5-dehydrogenase, MtlD, in a reversible reaction (Fig 1C) [35,36]. In *Escherichia coli* (amino acid sequence similarities with *S*Tm: MtlA: 95%, MtlD: 93%, MtlR: 91% [37,38]), the expression of *mtlA* and *mtlD* is controlled by the transcriptional repressor MtlR. MtlR activity is enhanced by interaction with the dephosphorylated phosphocarrier protein HPr, which accumulates during glucose transport [32]. Mannitol is thereby subject to catabolite repression: glucose inhibits the uptake of mannitol [39]. As in *E*. *coli*, the accumulation of the intermediate mannitol 1P in an *mtlD* knockout mutant is toxic for *S*Tm [40].

While mannitol is metabolized efficiently in bacterial monocultures growing on mannitol as the main carbon source, it is yet unclear whether mannitol is available and taken up by *S*Tm when growing intracellularly. To address this, we probed whether we could exploit the mannitol sensitivity of an *mtlD* knockout strain. In *S*Tm monocultures of the wild type (wt), *ΔmtlA*, or *ΔmtlD* (in MOPS medium with no host cells present), both Δ*mtlA* and Δ*mtlD* grew poorly on mannitol as the main carbon source (residual growth of Δ*mtlA* is due to amino acids present in the growth medium) (Figs 1D and S1C). In contrast, only Δ*mtlD* failed to grow on mannitol and glycerol (a non-repressive carbon source), confirming its extreme mannitol sensitivity (Figs 1D and S1C). This sensitivity was largely masked when mannitol was combined with glucose or glucose 6P (Figs 1D and S1B and S1C), as expected from glucose repression, and in line with previous experiments [39].

We then used the *mtlD* knockout mutant alongside the wt in an in vitro infection setup (gentamicin protection assay, see Methods) of RAW264.7 (Fig 1E) or HeLa cells (S1E Fig). We infected mammalian cells with a multiplicity of infection (MOI) of 100, treated them +/− mannitol, and isolated and counted bacteria at 20 h post-infection (hpi). Mannitol reduced the number of ∆*mtlD* bacteria by three orders of magnitude in RAW264.7 cells (Fig 1E) and by more than one order of magnitude in HeLa cells (S1E Fig). Hence, mannitol is metabolized by intracellular *S*Tm, despite the presence of glucose in the growth medium. Glucose repression is therefore not fully active in these conditions, possibly due to low intracellular glucose levels in the host cells.

To assess whether mannitol alters bacterial physiology compared to glucose, which is an essential nutrient during intracellular *S*Tm growth [41], we first measured the growth rate of *S*Tm in the two carbon sources, which was indistinguishable (S1F Fig). Secondly, we quantified metabolite levels of *S*Tm monocultures growing in the two carbon sources and did not find significant changes for any of the 67 intracellular metabolites quantified, except for mannitol 1P (S1G Fig). Hence, mannitol and glucose result in very similar physiology for *S*Tm. To assess if mannitol affects *S*Tm physiology when *S*Tm is growing intracellularly in the host, we tested whether mannitol would prevent the uptake of other carbon sources into *S*Tm during intracellular infection, which could greatly skew the intracellular *S*Tm metabolism. Using a *glpD* knockout mutant that grows worse when glycerol is taken up into *S*Tm in combination with a non-repressive carbon source (“sugar-phosphate toxicity” [42], S1D Fig), we confirmed that this glycerol-induced growth defect was not suppressed during intracellular infections by mannitol (S1H Fig), which is a repressive carbon source in batch culture (S1D Fig). Overall, we concluded that the presence of mannitol has a very similar effect on the intracellular *S*Tm metabolism as glucose, and it does not repress the uptake of other carbon sources intracellularly.

To further examine the effect of mannitol on host cells, we compared intracellular metabolite levels in RAW264.7 and HeLa cells, with or without the addition of mannitol for 12 h. None of the quantified metabolites were more than 2-fold different between the two conditions (adjusted *p*-value <0.05), except for mannitol and mannitol 1P (S1I and S1J Fig), with the latter being likely produced by a nonspecific mammalian enzyme. Thus, we considered mannitol a suitable carbon source to probe bacterial intracellular metabolism: It traverses across mammalian host cells and reaches the intracellular compartments where *S*Tm resides during infection without confounding the native metabolism.

### Incorporation of ^13^C into *S*Tm metabolism can be robustly quantified

**Fig 2:**
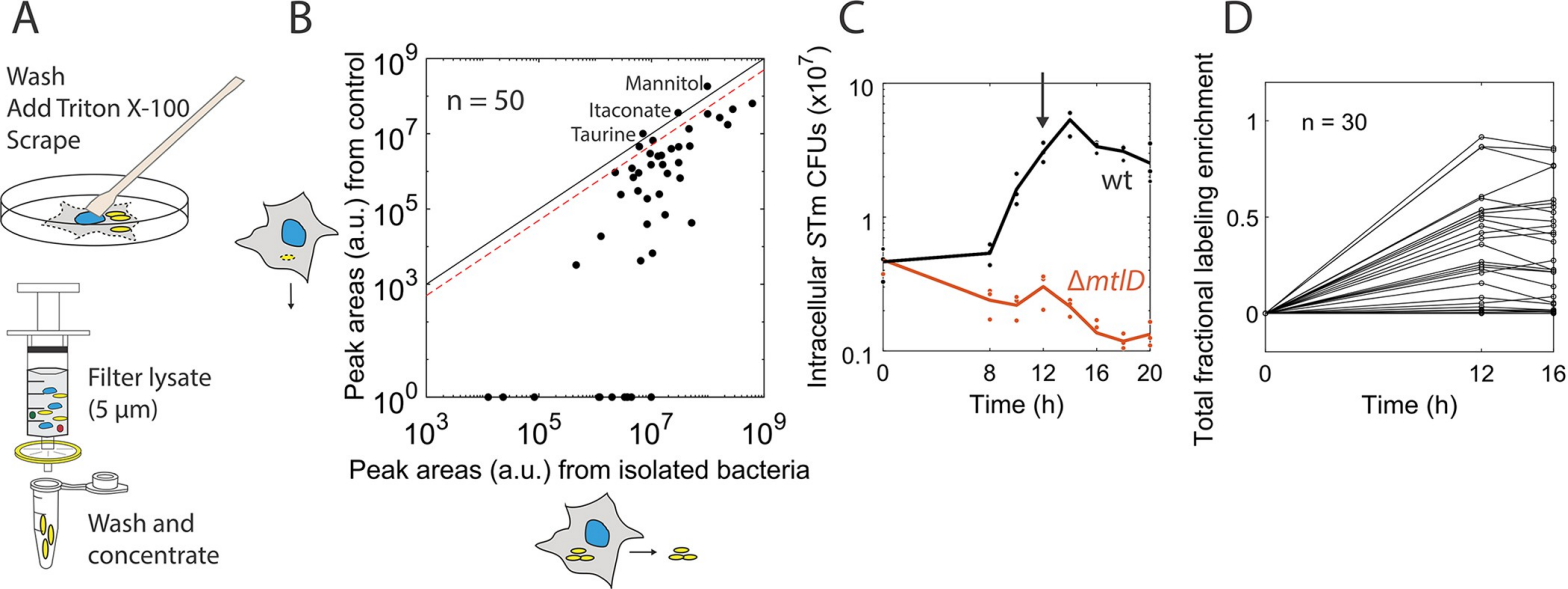
Bacterial isolation and optimal sampling time point allow the robust quantification of bacterial ^13^C labeling. (A) Bacterial isolation protocol: Infected host cells were lysed by the addition of ice-cold Triton X-100 in 0.9% NaCl, scraped from the cell culture plates, and the lysate was filtered through a 5 μm filter on ice; bacteria were washed and concentrated by centrifugation. (B) Metabolite peak areas (a.u.) for 50 metabolites from a sample after the bacterial isolation protocol (x-axis), compared to the control where RAW264.7 cells were infected with heat-inactivated, non-replicating *S*Tm, processed in the same way (y-axis). Both samples were from 12 hpi with MOI 100. The black line indicates equal peak areas in the sample and control; the red dashed line indicates 2-fold peak areas in the sample versus control. For most metabolites, the sample has a >2-fold signal over the control. Data are representative of 2 independent experiments. (C) *S*Tm CFUs during infection of RAW264.7 macrophages for *S*Tm wt and Δ*mtlD* in a mannitol-containing medium. The arrow shows the chosen 12 hpi time point for metabolomics sampling. Lines depict the average of biological triplicates. (D) TFLE compared between 12 hpi and 16 hpi of RAW264.7 macrophages for 30 metabolites from 1 experiment (mean from biological duplicates). The data underlying this figure can be found in S1 Data. a.u., arbitrary unit; CFU, colony-forming unit; hpi, hours post-infection; MOI, multiplicity of infection; *S*Tm, *Salmonella* Typhimurium; TFLE, total fractional labeling enrichment.

Since bacterial cells comprised only approximately 2.3% of the total cellular material (host cells and bacteria, S2A Fig), quantification of the unlabeled fraction of bacterial metabolites is largely contaminated by their host counterparts. To reliably analyze bacterial labeling patterns, we, therefore, needed to separate the bacteria from the host. After host cell lysis, we filtered the bacteria-containing cell lysate through a 5 μm filter, through which bacteria should pass, but most host cell organelles not [43] (Fig 2A), and isolated, washed, and concentrated the bacteria in the flow-through by a two-step rapid centrifugation. Thereby bacterial proteins were enriched by a factor of 50 (S2B Fig), and mitochondrial proteins by a factor of 3, whereas host nuclear, cytosolic, and Endoplasmic Reticulum (ER) proteins were depleted by a factor of >5 in RAW264.7 cells (S2B Fig). Enrichment of *S*Tm from HeLa cells was less pronounced, but the depletion of host proteins was at least as effective (S2C Fig). We concluded that this experimental step offered a rapid way to enrich for bacteria.

As host cell organelles, especially mitochondria, could not be removed completely (S2B Fig), we quantified whether host cell metabolites would substantially contaminate our signal by comparing metabolite levels between infected and mock-infected (heat-inactivated *S*Tm) RAW264.7 cells after our isolation protocol. RAW264.7 cells still react to heat-inactivated bacteria and show phenotypic similarities to cells infected with live bacteria [44]. However, heat-inactivated bacteria are dead and are cleared by the host. Hence, any metabolites extracted from this mock-infected control using our isolation protocol reflect contamination by the host cells. The big majority of the 50 quantified metabolites (90%) had at least 2-fold higher levels in cells infected with live bacteria (Fig 2B). The only metabolites with similarly high levels in the control were mannitol, taurine, and itaconate: Mannitol is present at high levels in the cell culture medium, taurine is ubiquitous in cells [45], and itaconate is produced by macrophages stimulated by lipopolysaccharide (LPS) [46]. For all metabolites for which we quantified and interpreted the fractional ^13^C enrichment in subsequent experiments (S1 Table), the contaminating fraction from host cells accounts for at most 20% (S2D Fig). Hence, host contamination does not play a major role in the metabolites we quantify.

Our enrichment protocol for bacteria during infection (Fig 2A) is effective, but it takes approximately 20 min. During this time, metabolic labeling patterns may change—e.g., because of the activation of some metabolic pathways. To assess the impact of our isolation protocol on labeling patterns, we used bacterial monocultures grown in MOPS medium on 50% U-^13^C mannitol and compared our established isolation protocol (“standard protocol”) to a filtering isolation protocol (“fast protocol,” approximately 3 min, Methods). In case the labeling varies significantly between the two protocols, data for such a metabolite must be interpreted with care as its labeling enrichment is likely affected by the time-delay of the isolation procedure. We found the fractional ^13^C enrichment to be relatively stable (S2E Fig): Out of 38 quantified metabolites, only 8 (related to lower glycolysis and the tricarboxylic acid (TCA) cycle) showed significantly lower total fractional labeling enrichment (TFLE = 1 – unlabeled fraction) in the fast protocol, implying that more label was incorporated into these metabolites in the standard protocol. In contrast, metabolites from upper glycolysis, the pentose phosphate pathway (PPP), amino acids, nucleotide, glutathione, and energy metabolism had highly conserved TFLEs (S2E Fig). In addition, the dominating mass trace (besides M+0) remained the same, even in metabolites with significantly different TFLE (examples in S2F Fig). Among the observed metabolites, only fumarate showed a very distinct pattern compared to the fast isolation protocol (S2G Fig). Therefore, we only used these 8 metabolites for qualitative data interpretations further on, and not as inputs to the quantitative ^13^C flux modeling. Overall, our protocol allows for isolation of bacteria from a lysate of infected host cells, while the fractional ^13^C enrichment stays stable for most metabolites. The high level of purity (Fig 2B) enabled us to analytically determine and interpret the exact fractional ^13^C enrichment of many bacterial metabolites, largely unbiased by the unlabeled host metabolites.

### Optimized sampling time enables model-assisted data interpretation

To allow a model-assisted interpretation of the emerging complex labeling patterns in the host–pathogen co-metabolism, we looked for an optimal sampling time supporting metabolic and isotopic steady state. Metabolic steady-state conditions are, however, impossible under cell culture infection conditions, as nutrient levels change and heterogeneity of intracellular *S*Tm is well established [47,48]. To approach a pseudo-steady-state setting, which is common in adherent cell culture labeling studies [49], we used conditions in which extracellular nutrients were plentiful, and their concentrations changed relatively slowly. By following the infection of host cells with wt *S*Tm for 20 h, we determined a window between 8 and 14 h in which the bacterial cell numbers increased approximately exponentially in both RAW264.7 and HeLa cells (Figs 2C and S2H). Cell numbers of the Δ*mtlD* mutant decreased at our first sampling time point, 8 hpi, indicating that mannitol reached the intracellular bacteria early during the infection (Fig 2C). Monitoring absolute nutrient concentrations in the mammalian cell culture medium of RAW264.7 cells indicated that all nutrients were depleted slowly, except for glucose, which was used up at 12 hpi. In contrast, for HeLa cells, all nutrients including glucose were depleted slower (S2I Fig). Thus, we chose the 12 hpi for sampling both cell lines, controlling that there was still glucose present in the RAW264.7 cell cultures at the end of the experiment.

We then checked whether and when the labeling enrichments of intracellular *S*Tm metabolites are close to their isotopic steady states under the studied infection conditions. An approximately equilibrated isotopic labeling state allows the interpretation of isotopologues without the knowledge of metabolite concentrations. To ascertain approximate isotopic stationarity—whereby changes in the fractional isotope incorporation in the quantified metabolites are sufficiently low to allow estimating fluxes—we compared the labeled fraction at a later time point (16 hpi) with that of the 12 hpi: Most metabolites had highly similar TFLEs (Fig 2D) and correlated fractional ^13^C enrichments (r = 0.997) (S2J Fig). This confirms a metabolic and isotopic pseudo-stationarity at 12 hpi. As our method cannot capture subpopulations, known to be present during *S*Tm replication in macrophages [47,48], the choice of the relatively late 12 hpi time point increases the chance that the data represent the metabolic state of actively replicating *S*Tm.

### Isotope labeling reveals active *S*Tm pathways and flux directionalities during infection

**Fig 3:**
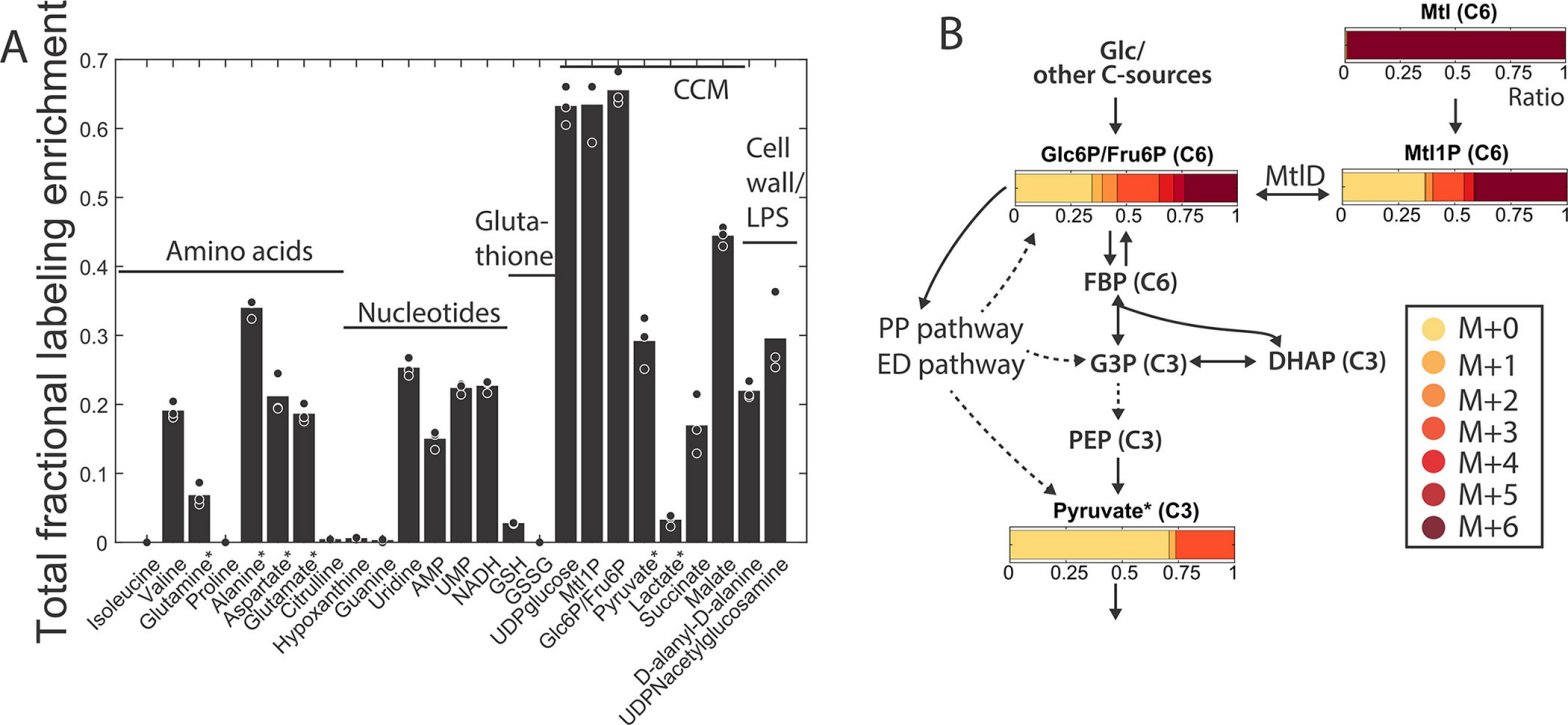
Bacterial metabolite labeling from U-^13^C mannitol. (A) Total fractional labeling enrichment (TFLE = 1 – unlabeled fraction) of different bacterial metabolites isolated from RAW264.7 cells at 12 hpi with MOI 100. Bars show averages of biological triplicates. CCM: central carbon metabolism, GSH: reduced glutathione; GSSG: oxidized glutathione. (B) Fractional ^13^C enrichment (long axis of bars, “Ratio”) of glycolytic bacterial metabolites isolated from the same experiment as (A) for the different isotopologues (M+0, M+1, M+2, …). U-^13^C mannitol (mtl) on the top right of the plot is the source for all labels in the isotopologues with higher masses (M+1, M+2, …). Dashed arrows depict lumped reactions. Note that glucose (glc) 6P and fructose (fru) 6P are indistinguishable by our analysis. Data are corrected for the natural ^13^C abundance and the ^12^C isotopic impurity of U-^13^C mtl and depict the average of biological triplicates; in (A) and (B) metabolites quantitatively affected by the enrichment protocol (S2E Fig) are denoted with *. The data underlying this figure can be found in S1 Data. DHAP, dihydroxyacetone phosphate; ED, Entner–Doudoroff; FBP, fructose 1,6 bisphosphate; G3P, glyceraldehyde 3P; hpi, hours post-infection; LPS, lipopolysaccharide; MOI, multiplicity of infection; mtl, mannitol; PEP, phosphoenolpyruvate; PP, pentose phosphate.

To identify metabolic pathways that are actively used during infection, we isolated *S*Tm at 12 hpi (standard isolation protocol) from RAW264.7 macrophages and HeLa cells (cultivated in the same host cell medium) and determined the TFLEs of 25 confidently detected bacterial metabolites (see Methods, S1 Table) across several metabolic pathways: central carbon metabolism including the TCA cycle, glutathione, and nucleotide metabolism, NAD metabolism, metabolism of 1 non-proteinogenic, and 7 proteinogenic amino acids, and cell wall/LPS biosynthesis (Fig 3A). As the host cells cannot degrade mannitol (Figs 1B and S1A), all labeled metabolites were produced de novo by intracellular bacteria. The quantified fractional labeling enrichment from these 25 metabolites was highly reproducible between biological replicates (r = 0.98; S3A Fig). The TFLE ranged from 0% (isoleucine, proline, hypoxanthine, guanine) up to 60% (glucose 6P/fructose 6P, mannitol 1P, UDP-glucose) (Fig 3A), meaning that *S*Tm takes up other unlabeled carbon sources in addition to mannitol. Overall, the same metabolites were labeled in RAW264.7 and HeLa cells, with HeLa cells showing overall less labeling (S3B Fig), in line with the less pronounced mannitol metabolism of intracellular *S*Tm in HeLa cells (see Figs 1E and S1E). Valine and succinate have specifically high labeling in RAW 264.7 cells. Succinate has recently been shown to accumulate in host cells during intracellular *S*Tm infection and to promote virulence in *S*Tm [50]. Nitric oxide stress in macrophages, but not in HeLa cells [51], is known to activate valine biosynthesis in *S*Tm [52], explaining the higher fractional enrichment of valine in RAW264.7 cells. Overall, the active pathways overlapped with those previously identified by mutant studies as relevant for *S*Tm intracellular replication. In particular, mutants in glycolysis [41], nucleotide biosynthesis [9], and cell wall biosynthesis [8] are known to be strongly defected in macrophages. On the other hand, *de novo* production of glutathione and citrulline have not been previously described under infection conditions.

To refine our insights into metabolic activities, we determined the percentage of the total metabolite pool for each specific metabolite that is unlabeled (M+0), has one heavy (^13^C) carbon atom (M+1), two (M+2), three (M+3), and so on, focusing on *S*Tm isolated from RAW264.7 cells. Although the mass isotopomer distributions (MIDs) are available, there is no positional labeling information. To accurately interpret MIDs, not only the net flux (i.e., the difference between the forward and backward flux of a reaction), but also the simultaneous action of forward and backward fluxes need to be considered [53]. This is exemplified by the labeling pattern of mannitol 1P (Fig 3B): mannitol 1P has an M+6 trace, but also a strong M+0 and M+3 trace, similar to glucose 6P/fructose 6P. Here, it is obvious that MtlD must exchange isotopes, harmonizing the MIDs of mannitol 1P and glucose 6P/fructose6P. This backward operation of MtlD in the acidic vacuole is in line with previous in vitro experiments that have shown that the backward reaction is highly active under acidic conditions [36]. Reaction bidirectionality can be due to a bidirectional enzyme (such as MtlD) or due to the opposite activity of two unidirectional enzymes (e.g., 6-phosphofructokinase (PfkA/B) for the forward and fructose-bisphosphatase (Fbp) for the backward reaction). Similarly, the surprising M+3 labeling in glucose 6P/fructose 6P (Fig 3B), coming from only uniformly labeled mannitol or unlabeled carbon sources as direct substrates, can be interpreted in different ways. One way is that fully labeled C6 bodies (from mannitol) and unlabeled C6 bodies (e.g., from glucose 6P) make their way through glycolysis until the C6 bodies are split into two C3 bodies (glyceraldehyde 3P and dihydroxyacetone phosphate), which are then combined and metabolized gluconeogenetically to form the observed labeling pattern in fructose 6P. Alternatively, the M+3 labeling in glucose 6P/fructose 6P may also be derived by contributions from the Entner–Doudoroff pathway (EDP) or the PPP (Fig 3B). To discern between the different theoretical possibilities, we decided to use the model-based approach of ^13^C MFA [15,54].

### Bayesian modeling reveals that backward glycolysis and the Entner–Doudoroff pathway are active, independently of glucose availability

To infer nutrient uptake and intracellular fluxes, we formulated a reaction network of the central carbon metabolism of *S*Tm consisting of glycolysis (Embden–Meyerhof–Parnas pathway), PPP, EDP, TCA cycle, anaplerosis, nucleotide biosynthesis (including NADH biosynthesis), as well as the synthesis of specific amino acids and the nucleotide sugar UDP-N-acetylglucosamine (S4 Fig). In addition to the mannitol uptake step, uptake reactions for other potential substrates were included (e.g., sugars, nucleobases, amino acids; Methods). For the analysis of MIDs, we pruned the model by excluding pathways downstream of pyruvate due to quantitative unreliability in the labeling patterns of TCA metabolites (S2E Fig and Methods). Compared to the conventional ^13^C MFA setting [53], the many potential carbon entry points (in total 18) represent a challenge for flux inference. We, therefore, used Bayesian model averaging (BMA)-based ^13^C MFA, a rigorous statistical multi-model approach, which is suited to deal with heavily underdetermined systems [55]. As a result, BMA-based ^13^C MFA provides the joint net flux posterior probabilities for all intracellular and uptake reactions, along with their credible intervals, and, thereby, the dominating reaction directions for all reactions (S2 Table). This enabled us to identify which of the potential carbon sources are likely metabolized: If the 95% credible intervals of the uptake fluxes exclude the zero value, we concluded that the respective metabolite is taken up by *S*Tm.

**Fig 4:**
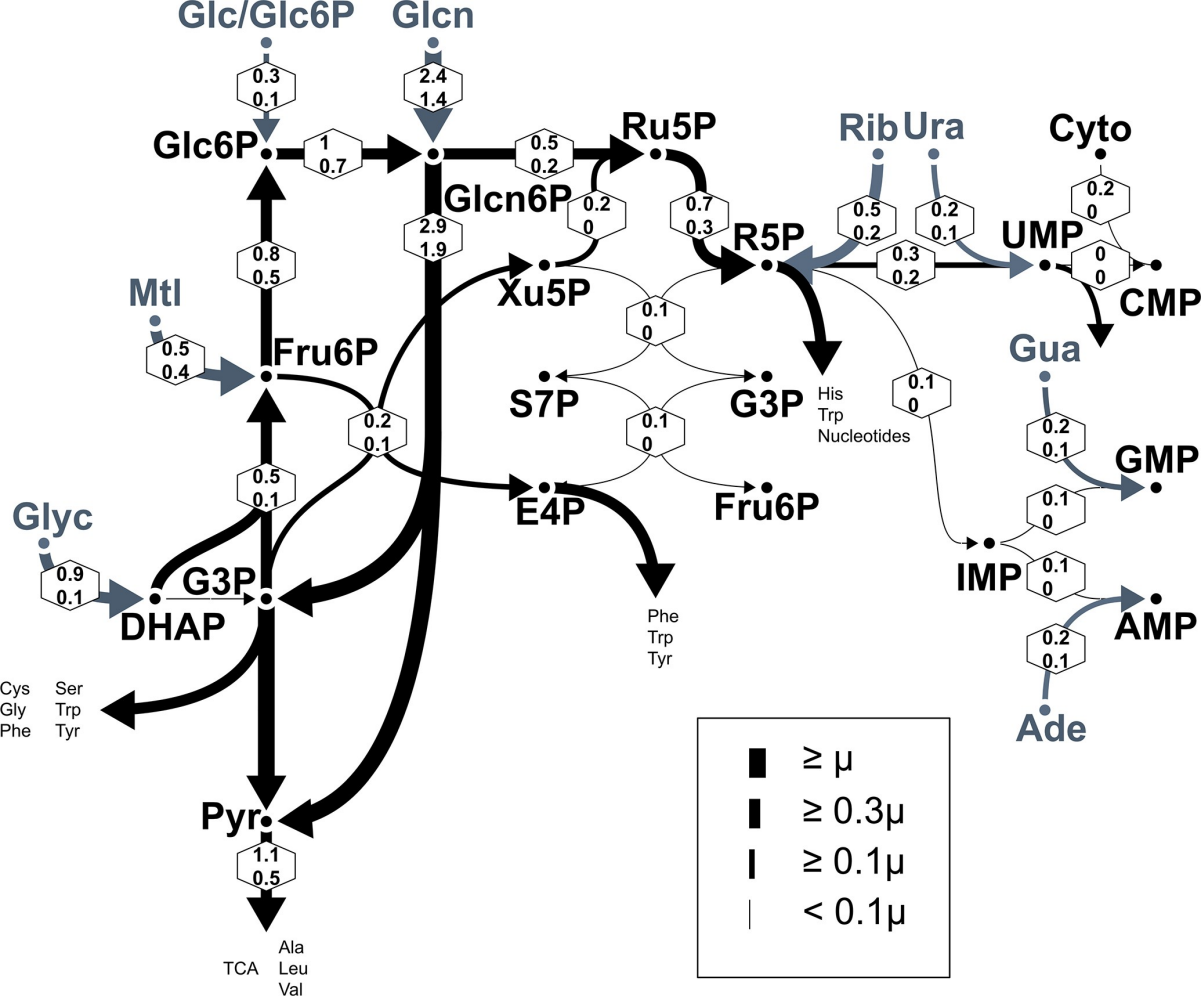
Summarized flux map for the intracellular *S*Tm metabolism inferred by BMA-based ^13^C MFA. Net flux 95% credible intervals (in hexagons) were estimated from labeling data (low glucose condition), where the thickness of the arrow indicates the expected value (see also S5A Fig). Values are given relative to the growth rate (μ). Arrowheads denote the dominating reaction direction. Gray labels/arrows depict nutrient uptakes. Glc, glucose; Glc6P, glucose 6 phosphate; Glcn, gluconate; Glyc, glycerol; Pyr, pyruvate; Ru5P, ribulose 5P; R5P, ribose 5P; Xu5P, xylulose 5P; S7P, sedoheptulose 7P; E4P, erythrose 4P; Rib, ribose; Ura, uracil; Cyto, cytosine; Gua, guanine; Ade, adenine; amino acids with standard 3-letter abbreviations. A visual representation of the complete model used for the analysis is in S4 Fig; the formal specification of the ^13^C metabolic model is found in the S1 File. The data underlying this figure can be found in S1 Data. BMA, Bayesian model averaging; MFA, metabolic flux analysis; *S*Tm, *Salmonella* Typhimurium.

Our BMA-based ^13^C MFA revealed that besides mannitol, the main carbon sources taken up by *S*Tm when inside macrophages are gluconate, glycerol, ribose, and glucose/glucose 6P (Figs 4 and S5A). Out of these, only glucose and mannitol are part of the medium (Methods). The rest should thus come from the host. The uptake and co-utilization of the various sugars are in line with previous reports [4,7,41]. In particular, gluconate was detected inside the host cells, consistent with its availability to *S*Tm (S3 Data). Interestingly, from glycerol and mannitol, the main carbon flow is upwards, and together with gluconate, is directed towards the EDP, before entering lower glycolysis and downstream metabolic pathways. The EDP is an alternative glycolytic pathway that, while supplying less energy, comes at lower costs in terms of the enzymes needed [56], and is active under chemostat conditions at slow growth rates [57]. Our data demonstrate that the EDP becomes the major glycolytic pathway during *S*Tm intracellular growth, in accordance with prior gene expression measurements during *S*Tm infection [4,58].

Having the flux map at hand, we traced the origin of the M+3 isotopologue in glucose 6P/fructose 6P. The backward direction of the upper glycolysis (from glyceraldehyde 3P to fructose 1,6-bisphosphate to fructose 6P and glucose 6P) is indeed the primary metabolic direction (Fig 4 and S2 Table). However, there is insufficient evidence that the glycolytic forward direction is simultaneously active (probability for the forward direction being approximately 40%) (S2 Table). As the TalA/B flux (non-oxidative PPP) is very low, the M+3 isotopologue of glucose 6P/fructose 6P is mostly generated through the EDP and backward glycolysis.

Metabolism is very sensitive to extracellular conditions [49]. To ascertain that our analysis provides relevant insights for infection conditions, we generated another data set with a slightly different nutrient input: keeping all other concentrations the same, we increased the (unlabeled) glucose concentration in the host cell medium resulting in an approximately 3-fold increase at 12 hpi (S5B Fig). The higher glucose concentration led to a lower fractional ^13^C enrichment overall (S5C Fig), as expected from the smaller proportion of uniformly labeled (U-^13^C mannitol) carbon source present, but MIDs were overall very similar (S4 Data). We then analyzed the labeling data set for the higher glucose concentration using the same model as before and BMA-based ^13^C MFA. Despite the differing glucose concentrations in the host nutrient composition, the net fluxes relative to the growth rate of the pathogen were very similar to our previous experiment with the lower glucose concentration (S5A Fig). Minor differences in the flux maps were a relative decrease in the uptake of mannitol and increased uptakes of glucose/glucose 6P and ribose, accompanied by an increased flux towards the oxidative PPP.

Overall, our labeling data together with the network-based quantitative analysis revealed the major active pathways, such as EDP, glycolysis operating in the backward direction, and the uptakes of gluconate, glycerol, ribose, and glucose/glucose 6P. Importantly, these findings were consistent between the two media conditions, corroborating our approach, and suggesting that intracellular *S*Tm proves to be relatively insensitive to small changes in the host milieu (in terms of net fluxes relative to the specific growth rate).

### Intracellular *S*Tm produces the ribose part of nucleotides de novo, whereas it takes up nucleobases from the host

**Fig 5:**
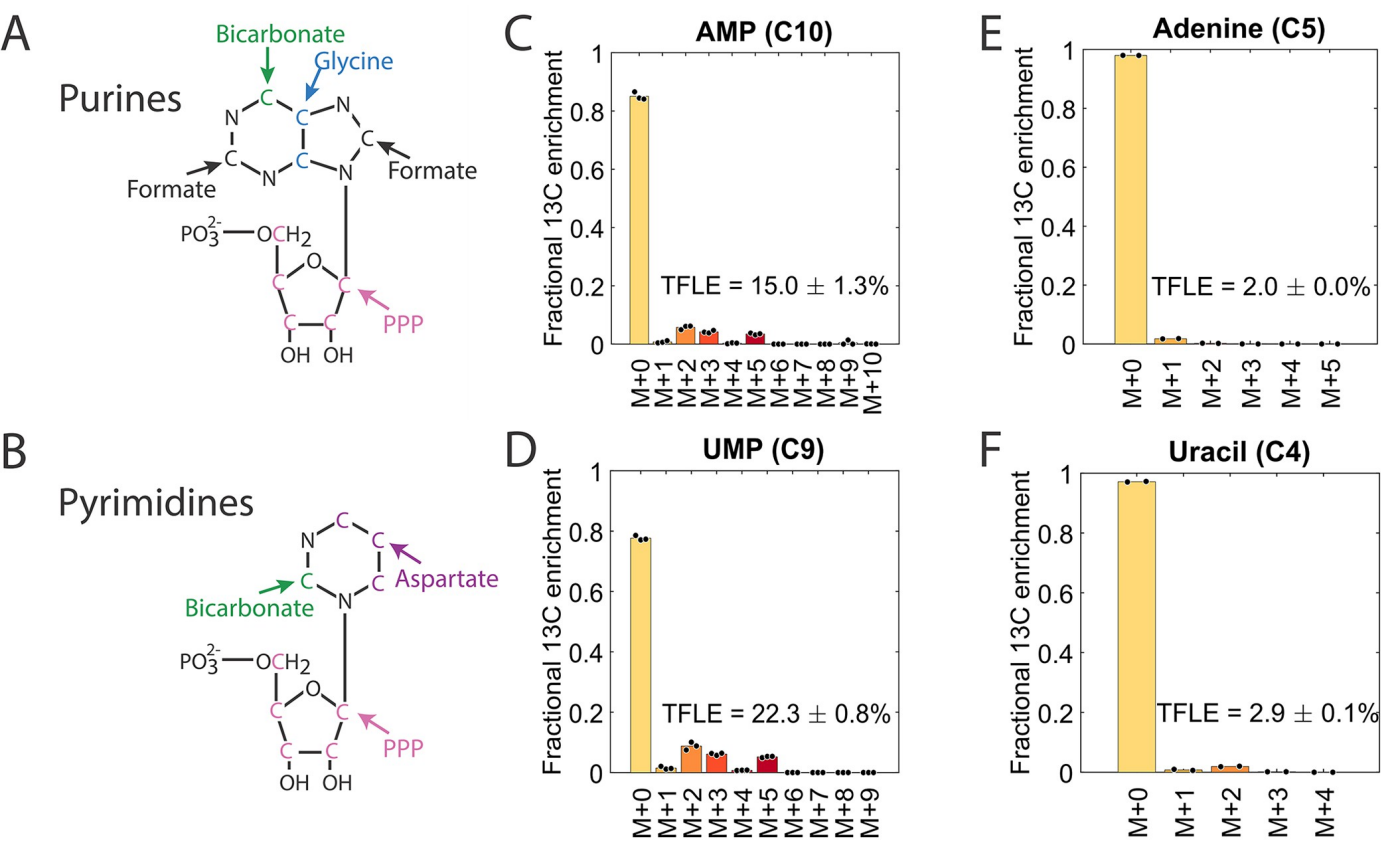
Purines and pyrimidines have only their ribose unit labeled. (A) Purine base structure and origin of C-atoms. The ^13^C-labeled C-atoms theoretically stem from formate, glycine, bicarbonate, or the ribose unit via the PPP. (B) Pyrimidine base structure and origin of C-atoms. The ^13^C-labeled C-atoms theoretically stem from aspartate, bicarbonate, and the ribose unit via the PPP. (C, D) Fractional ^13^C enrichment of AMP and UMP (nucleotides, with the ribose units) in bacteria isolated from RAW264.7 macrophages. Bars are averages of biological triplicates. Data are corrected for the natural ^13^C abundance and the ^12^C isotopic impurity of the uniformly labeled ^13^C mannitol. (E, F) Fractional ^13^C enrichment of adenine and uracil (without the ribose units) in bacterial RNA isolated from RAW264.7 macrophages. Bars are averages of biological duplicates. The data underlying this figure can be found in S1 Data. PPP, pentose phosphate pathway; TFLE, total fractional labeling enrichment.

The BMA-based ^13^C MFA model also enabled us to disentangle which proportion of a specific metabolite stems from assimilated carbon and which proportion is synthesized de novo. This question is particularly interesting for nucleotide biosynthesis, which is essential for intracellular *S*Tm [8,9]. The precursors for nucleotides are ribose 5P and the nucleobases, which are produced by bicarbonate, glycine, and formate for purines and bicarbonate and aspartate for pyrimidines (Fig 5A and 5B). We found that ribose 5P is only partly derived from assimilated ribose, whereas the majority (approximately 60% of the total) was synthesized de novo. De novo synthesis occurs via two routes, the forward-directed oxidative PPP (main route) and the reductive PPP operating in reverse direction via transketolase TktA2/B2 and Rpe (Figs 4 and S4). In contrast, the nucleobases are mostly assimilated by *S*Tm: for uracil there is still partial synthesis, whereas for adenine and guanine, nearly no de novo biosynthesis occurs (Fig 4).

To independently validate this result, we isolated bacterial RNA at 20 hpi and hydrolyzed it to obtain nucleobases (Methods). We measured the fractional ^13^C enrichment by LC–MS and compared it to ribonucleotide pools (Fig 5C–5F). While the ribonucleotides showed distinct and consistent labeling pattern in M+2, M+3, and M+5 (Figs 5C and 5D and S6), the nucleobases alone were minimally labeled (Fig 5E and 5F), confirming the modeling. At this point, we cannot distinguish whether nucleobases are taken up as they are from the host or whether they are also partially assembled from unlabeled precursors (e.g., orotate for pyrimidines).

### Phosphoenolpyruvate carboxylase is key during intracellular infection

**Fig 6:**
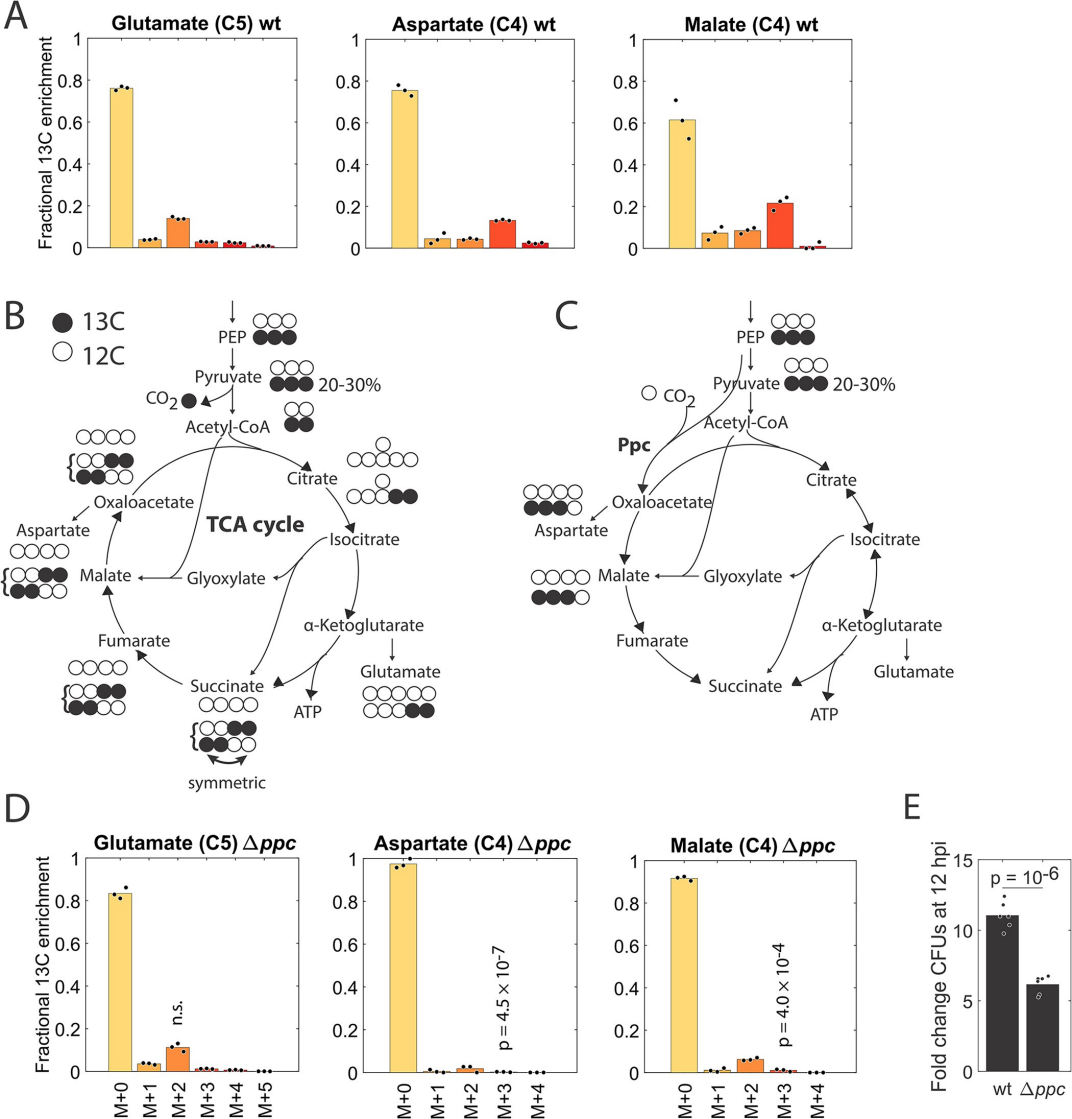
Ppc underlies the strong M+3 labeling in the TCA cycle and is required for intracellular *S*Tm replication. (A) Mass isotopomer distributions of glutamate, aspartate, and malate in the wt. (B) If a labeled PEP/pyruvate (labeling percentage is approximately 20%–30%, see Fig 3B) proceeds through pyruvate dehydrogenase, combining with unlabeled oxaloacetate, it generates an M+2 trace in citrate, isocitrate, α-ketoglutarate, and glutamate, etc. in the first round of the TCA cycle. The symmetry in succinate could result in M+3 and M+4 labeled α-ketoglutarate and downstream metabolites when M+2 labeled oxaloacetate combines with labeled acetyl-CoA in further rounds of the TCA cycle. Due to the initially low labeling percentage in pyruvate, however, substantial percentages of M+3/M+4 labeled metabolites are impossible. (C) If a labeled PEP proceeds through Ppc, combined with an unlabeled CO_2_, it can generate a substantial M+3 trace in oxaloacetate, aspartate, malate, and downstream metabolites. As the reaction from succinate to α-ketoglutarate is irreversible, the M+3 trace cannot, however, proceed to α-ketoglutarate and glutamate. (D) Mass isotopomer distributions in a *ppc* knockout. *p*-values for the M+2 trace in glutamate and the M+3 trace in aspartate and malate compared to the wt are from a two-sample *t* test. (E) Fold change in CFUs 12 hpi in the wt and ∆*ppc* mutant. The *p*-value is from a two-sample *t* test. For A, D, E: Bars are averages from 3–6 replicates. The data underlying this figure can be found in S1 Data. CFU, colony-forming unit; hpi, hours post-infection; PEP, phosphoenolpyruvate; *ppc*, phosphoenolpyruvate carboxylase; *S*Tm, *Salmonella* Typhimurium; TCA, tricarboxylic acid; wt, wild type.

As our bacterial isolation protocol quantitatively affects the MIDs of TCA cycle metabolites (S2E Fig), this did not allow for a model-based quantitative analysis of the TCA. Nonetheless, we could derive qualitative hypotheses about the (in)activity of TCA/anaplerotic reactions (S1 Table). Strikingly, some labeling patterns in successive steps of the TCA cycle were discordant (Fig 6A). For example, glutamate had a pronounced M+2 trace, which likely originated from acetyl-CoA produced by the pyruvate dehydrogenase complex (Fig 6B). In contrast, aspartate and malate (and to some extent also succinate, S7A Fig) had a pronounced M+3 trace (Fig 6A). An M+3 labeling in malate and aspartate could in principle be generated by further rounds of a fully running TCA cycle by combining an M+2 labeled oxaloacetate with an M+2 labeled acetyl-CoA (Fig 6B). However, as pyruvate is only labeled at 20% to 30% (Fig 3B), the chance to get such M+3 or M+4 labeling is low and consequently is unable to explain the >20% M+3 labeling in malate. In addition, in that case, we should also see a strong M+3 signature in glutamate, which is factually absent (Fig 6A). Also, the glyoxylate shunt cannot create such an M+3 labeling in malate and aspartate. A different explanation for the M+3 labeling in malate is the activity of the phosphoenolpyruvate carboxylase (Ppc), a reaction that was suggested before to be relevant for intracellular *S*Tm [27]: here, a fully labeled PEP combines with an unlabeled CO_2_ to M+3 labeled oxaloacetate (Fig 6C). Ppc is an anaplerotic reaction that is also part of the mixed acid fermentation path from pyruvate to succinate in *S*Tm [59].

To experimentally confirm the contribution of Ppc, we determined the labeling in glutamate, aspartate, and malate in a *ppc* knockout mutant and the wt. Overall, the 2 exhibited similar labeling patterns, as judged by the TFLE of 32 metabolites (S7B Fig). While the M+2 labeling in glutamate was unaltered, the M+3 labeling in aspartate and malate turned into M+0 labeling in the *ppc* mutant (Figs 6D and S7C), confirming that the anaplerotic Ppc reaction is causal for the observed M+3 labeling in the 2 metabolites. A *ppc* knockout mutant showed approximately 50% reduction in CFUs after 12 hpi infection in RAW 264.7 macrophages, suggesting the importance and inescapability of this reaction for intracellular *S*Tm proliferation in our conditions (Fig 6E).

The anaplerotic Ppc reaction in *E*. *coli* is known to be essential for growth on single glycolytic carbon sources entering upper/lower glycolysis or the EDP, but not for growth on TCA cycle intermediates [60–62]. We experimentally confirmed the essentiality of Ppc in M9 glucose medium without amino acids in *S*Tm (S7D Fig), in agreement with previous studies [63]. In contrast, the *ppc* mutant grew nearly like the wt in rich LB medium (S7D Fig). The importance of Ppc for *S*Tm during intracellular growth is therefore explained by its predominant use of carbon sources that enter glycolysis and the EDP (Fig 4). Overall, our data underline the usage and key role of the anaplerotic Ppc reaction during infection.

## Discussion

Measuring direct metabolic activities of intracellularly replicating bacteria has not been possible until now, due to difficulties in distinguishing bacterial from host metabolites. Here, we provide a method to specifically trace bacterial metabolites during infection and use it to generate the first quantitative carbon flux map for an intracellular pathogen, *Salmonella*, growing inside macrophages. As a result, we confirmed prior results stemming from indirect measurements of metabolic activity [4,7–9] and contributed new findings on intracellular *S*Tm metabolism. In addition to revealing the active parts of metabolism during infection (glycolysis, TCA cycle, nucleotide, and NADH metabolism, biosynthesis of proteinogenic and non-proteinogenic amino acids, glutathione metabolism, and the biosynthesis of cell wall building blocks), we were able to reveal the major substrates *S*Tm feeds on and the main catabolic routes (foremost the EDP). Despite not being able to model anaplerosis, we showed that the anaplerotic phosphoenolpyruvate carboxylase reaction (ppc) is key during intracellular growth.

Overall, our data directly support the view that intracellularly proliferating *S*Tm receives and co-utilizes a plethora of different nutrients from the media and/or the host [7]. *S*Tm therefore only partially relies on the availability of the supplemented mannitol, largely feeding on other substrates. This highlights that the chosen mannitol-based approach while being artificial, does not perturb the metabolism significantly. Interestingly, nutrients like mannitol or glycerol that are normally repressed by glucose [39,64] are readily metabolized during intracellular *S*Tm growth. Although a higher glucose concentration in the host cell medium lowered the labeling from U-^13^C mannitol (S5C Fig), we believe that this reflects the decreased fraction of labeled substrate, not a repressive effect of glucose on mannitol. This absence/mitigation of glucose repression may be caused by very low steady-state glucose concentrations [65] (in the hundreds of μg/L), which may prevail inside the SCV [7]. While our data could not distinguish whether *S*Tm takes up glucose or glucose 6P, the latter has been suggested before to be excluded from the SCV, as the expression of its transporter UhpT can be used as a reporter for cytoplasmic *S*Tm [66].

Notably, *S*Tm growing inside macrophages or epithelial cells (grown in the same host cell medium) showed similar labeling patterns, suggesting that the *S*Tm metabolism is closely and directly interacting with the nutrients available in the host cell and the cell culture medium—without much active restriction by the host cells. Nutrients from the medium can reach *S*Tm via host cell/SCV membranes, but *Salmonella-*induced filaments have been shown to facilitate the uptake of nutrients through the host endosomal system [67]. Whether some of the imported metabolites are specifically facilitated by *Salmonella-*induced filaments needs further investigation. Bacteria may also secrete labeled metabolites into the host cells, which are then reimported again, but we consider their contribution to the overall fractional ^13^C enrichment insignificant, as the host metabolite pool is much bigger.

Our ^13^C MFA approach was able to infer relative fluxes despite uncertainties in the data. Establishing faster and more effective bacterial isolation protocols will further expand and improve flux estimations, allowing the application of our method to other cell types, primary cells instead of cell lines, or other host–pathogen and symbiotic interactions. Improved experimental setups with positionally labeled tracers like 1-^13^C mannitol [68], parallel labeling experiments [69], or isotopically nonstationary ^13^C MFA [70] could provide more informative flux estimations. Other carbon sources with similar properties like mannitol, i.e., they are taken up but not metabolized by host cells and incorporated into the bacterial metabolism, may provide additional insights into the bacterial metabolism. In addition, using mutants of specific steps in the metabolic pathways, as we did here with *ppc*, may help further to distinguish between the utilization of specific pathways [71]. While we decided on a broad LC–MS method that covers metabolites from central carbon metabolism, amino acids, and nucleotide metabolism, applying different (targeted) metabolomics techniques can provide more refined information on the activity of other parts of metabolism, like the fatty acid metabolism, or fermentative pathways [72]. Overall, the method presented here opens new avenues to further investigate the intracellular metabolism and physiology of *S*Tm or other pathogens that replicate inside host cells. Characterizing active and important metabolic pathways during pathogen intracellular growth could in the future inform the development of novel metabolism-based therapeutic strategies to inhibit difficult-to-treat pathogens.

## Materials and methods

### Strains, cell lines, and growth conditions

The mammalian cell lines RAW264.7 (ATCC TIB-71) and HeLa (ATCC CCL-2) were kept at 37°C and 5% CO_2_. RAW264.7 and HeLa cells were cultured in DMEM medium with 4.5 g/L glucose (Thermo Fisher Scientific, #41965039) containing 10% heat-inactivated fetal bovine serum (FBS, Sigma-Aldrich F9665) and passaged regularly using accutase (Thermo Fisher Scientific #A1110501) for RAW264.7 cells and trypsin-EDTA (Thermo Fisher Scientific 25300054) for HeLa cells. Only cells below a passage number of 15 were used for all experiments. For infection experiments, cells were seeded and treated in a medium with 1 g/L glucose and 0.11 g/L sodium pyruvate (Thermo Fisher Scientific, #11885084) containing 10% heat-inactivated (and dialyzed for metabolomics experiments) FBS (Sigma-Aldrich, **F0392**).

The bacterial wt used was *Salmonella enterica* subsp. Typhimurium 14028s and all knockout mutants (∆*mtlA*, Δ*mtlD*, Δ*ppc*, Δ*ssaV*) were generated by P22 transduction of the corresponding mutant from a single gene deletion library [73] into the wt and subsequent confirmation by PCR. The kanamycin concentration used for selection was 30 μg/mL. The primers used for checking were: ∆*mtlA*: CGCGACAGCAACATAAGAAGG and a primer binding inside the kanamycin resistance CAGTCATAGCCGAATAGCCT (k1); Δ*mtlD*: TGTGGAGAGGGTTAGGTTGAG and AGGCCTGGGTTTGTTCCATT; Δ*ppc*: CAGCAGTATTTCATGCCGCC and AGTAATTGACGCCACGGGTT; Δ*ssaV*: received from [74].

LB Lennox medium was used for bacterial cultivation unless stated otherwise. Heat-inactivation of *S*Tm was done by incubation in PBS at 65°C for 15 min (Figs 2B and S2D).

### Media, chemicals, and reagents

The following metabolite standards were purchased from Sigma-Aldrich: Amino acid standards physiological (A9906); D-glucose (Sigma Aldrich G8270); D-glucose-6-phosphate sodium salt (G7879); D-Fructose 6-phosphate disodium salt hydrate (F3627); D-Fructose 1,6-bisphosphate trisodium salt hydrate (F6803); Dihydroxyacetone phosphate lithium salt (37442); Phospho(enol)pyruvic acid monopotassium salt (P7127); Sodium pyruvate (P2256); Citric acid monohydrate (Roth 3958.2); DL-Isocitric acid trisodium salt hydrate (I1252); α-Ketoglutaric acid disodium salt hydrate (K3752); Succinic acid (S3674); Sodium fumarate dibasic (F1506); DL-Malic acid (240176); D-Ribose 5-phosphate disodium salt hydrate (R7750); Itaconic acid (I29204); γ-Aminobutyric acid (A2129); L-Alanyl-L-alanine (A9502); Uridine 5′-diphosphoglucose disodium salt hydrate (U4625); Uridine 5′-diphospho-N-acetylglucosamine sodium salt (U4375); β-Nicotinamide adenine dinucleotide, reduced disodium salt hydrate (N8129); N-Acetyl-L-glutamic acid (855642); Inosine (I4125); Cytidine (C4654); Uridine (U3750); Adenine (A2786); Thymine (T0376); Guanosine 5′-monophosphate disodium salt hydrate (G8377); Uridine 5′-monophosphate disodium salt (U6375); Adenosine 5’-monophosphate sodium salt (A1752); Guanosine 5′-diphosphate sodium salt (G7127); Cytidine 5′-diphosphate sodium salt hydrate (C9755); Uridine 5′-diphosphoglucose disodium salt hydrate (U4625); Adenosine 5′-diphosphate sodium salt (A2754); Guanosine 5′-triphosphate sodium salt hydrate (G8877); ATP (A16209); UTP (U6625); L-Glutathione oxidized (G4376); L-Glutathione reduced (G4251); Sodium L-lactate (L7022); Mannitol-1-phosphate lithium salt (92416), D-mannitol (M4125).

The following standards were obtained from MetaSci (Metasci, Toronto, Canada): Guanine, Shikimic acid, Glyceric acid, Pantothenic acid, Pyridoxine, Ascorbic acid, Aminobenzoic acid, Uracil, Indole, Sarcosine, Alanine, Taurine, Acetylphosphate, Folic acid, Glycerol-3-phosphate, Citrulline, Ornithine, Cis-aconitate, Pyroglutamic acid, gamma-Aminobutyric acid, cyclic Adenosine monophosphate, Guanosine, beta-Alanine. Note that not all of these metabolites could be detected in our samples.

Standards from other providers were: D-[UL-^13^C_6_]mannitol (Omicron, ALD-030) and amino acid standards (Supelco, A9906). As internal standards, we used Creatinine-(methyl-^13^C) (Sigma-Aldrich 488615) or an amino acid standard mix (Cambridge Isotope Laboratories, Inc., MSK-A2-1.2). Chemicals used in other experiments were: Gentamicin sulfate (Sigma-Aldrich G1914), Kanamycin disulfate salt (Sigma-Aldrich K1876); Triton X-100 (Sigma-Aldrich X100), amicase (Sigma Aldrich, A2427), glycerol (Geyer Th. 2050).

### Gentamicin protection assay

The gentamicin protection assay was performed as described before [75]. In brief, for RAW264.7 cell infections, cells were seeded 16 to 18 h before the infection in 6-well plates (0.9 × 10^6^ cells per well) or T-75 flasks (7 × 10^6^ per flask). *S*Tm were grown overnight with shaking at 37°C from a single colony in LB medium. For HeLa cell infections, cells were seeded in 6-well plates (0.2 × 10^6^ cells per well) or T-75 flasks (1.6 × 10^6^ per flask). *S*Tm were grown overnight with shaking at 37°C from a single colony in LB medium and subcultured (300 μL overnight culture in 10 mL LB Miller) for 3.5 h at 37°C, with shaking at 60 rpm. For RAW264.7 and HeLa infections, bacteria were washed once with PBS and resuspended in the same volume of PBS. Bacteria were added to the host cells at an MOI of 100, spun down at 170 g for 5 min at RT, and incubated for 25 min at 37°C to allow for bacterial uptake. Note that this MOI leads to cell death in around 30% of the host cells in 19 h [74], but released bacteria are quickly killed by gentamicin in the medium, and washed away before host cell lysis and bacterial isolation. Therefore, they do not contaminate the metabolic signals from the isolated alive bacteria. Cells were then washed once with DMEM medium containing 100 μg/mL gentamicin and incubated for 1 h with 100 μg/mL gentamicin in DMEM. After 1 h, the medium was exchanged for DMEM with 16 μg/mL gentamicin, and 4 g/L mannitol (U-^13^C-labeled or unlabeled) was added to the medium if applicable. This time point was defined as the time point zero (0 hpi). Cells were incubated at 37°C, 5% CO_2_ until the sampling time point.

### Quantification of bacterial intracellular replication

Mammalian cells were plated in 6-well plates (Nunc) 16 to 20 h before the infection. The cells were then subjected to a gentamicin protection assay as described above. At the sampling time point, the infected mammalian cells were lysed with 0.1% Triton X-100 in PBS and scraped from the plate. The lysate was diluted serially with PBS in 10-fold steps and 5 μL of each sample and dilution was pipetted in technical duplicates on square LB agar plates. The plates were incubated at 30°C or 37°C overnight and the bacteria were counted and photographed.

### Quantification of bacterial growth extracellularly

Pre-cultures were grown overnight shaking at 37°C in MOPS medium [76] containing 1 g/L amicase with 2 g/L glycerol, or 2 g/L glucose as the main carbon source. Samples were inoculated 1:100 from the glycerol pre-culture into fresh MOPS medium containing amicase with either 2 g/L glycerol, 2 g/L mannitol, or 2 g/L glycerol and 2 g/L mannitol. The additional samples were inoculated 1:100 from the glucose pre-culture into fresh MOPS medium containing amicase with either 2 g/L glucose, or 2 g/L glucose, and 2 g/L mannitol. For the experiments in S1D Fig, the pre-culture was grown in MOPS medium with 1g/L amicase and 2 g/L glycerol or 2 g/L sodium pyruvate and then diluted 1:100 into the final cultures analogously as described above. Absorbance at 600 nm was monitored every 10 to 15 min with continuous shaking at 37°C in a plate reader (Biotek Synergy HT) and the background-subtracted absorbance at 10 h after inoculation (the cultures had already reached stationary phase) was determined. For data in S7D Fig, cultures were pre-grown in LB or M9 glucose (1× M9 salts, 2 mM MgSO_4_, 0.1 mM CaCl_2_, and 2 g/L glucose) medium overnight, sub-diluted in the same medium, and measured every 15 min with continuous shaking at 37°C in the plate reader. All the data were processed and plotted using Matlab R2017b (The MathWorks). Growth rates (S1F Fig) were determined from the linear region on the background-subtracted log-transformed absorbance data.

Note that we considered “technical replicates” as parallel measurements from the same starting culture (e.g., growth of the same bacterial culture was measured in 2 wells in the plate reader); “biological replicates” as parallel measurements of different starting cultures (e.g., infection of multiple cell culture wells seeded the day before), and “independent experiments” as the repetition of the same or highly similar experiment on a different day with completely different starting cultures.

### Sample processing and metabolite extraction from mammalian cells

To test RAW264.7 or HeLa cells for their ability to metabolize mannitol (Figs 1B and S1A), the cells were seeded in 6-well plates (densities as above) in DMEM medium containing 1 g/L glucose, 0.11 g/L sodium pyruvate, and 10% FBS and supplemented with either 1 g/L uniformly U-^13^C-labeled glucose (Cambridge Isotope Laboratories CLM-1396-5), or 1 g/L uniformly U-^13^C-labeled mannitol; chemical purities are 99%. The cells were incubated at 37°C and 5% CO_2_ for 48 h, washed once with 10 mM ammonium acetate, quenched with 1 mL ice-cold 80% HPLC-grade methanol, and incubated at −80°C for 20 min. The cells were then scraped from the plates (kept on dry ice), vortexed thoroughly, and sonicated for 5 min in a cooled water bath. The cell debris was removed by centrifugation at 14,000 g for 10 min at 4°C. The supernatant was subsequently transferred to a new tube, dried under liquid nitrogen, and resuspended in 80% methanol for LC–MS analysis. The analysis was performed in biological triplicates.

To test whether the addition of mannitol affects the metabolism of host cells (S1I and S1J Fig), RAW264.7 and HeLa cells were grown as described above, with or without the addition of 4 g/L mannitol for 12 h. Cells were washed once with PBS, quenched with 1 mL ice-cold 80% HPLC-grade methanol, and incubated at −80°C for 20 min, and processed as above but with resuspension in a (Methanol-Acetonitrile-Water) MeOH:ACN:H_2_O (40:40:20) mixture. The analysis was performed on 6 biological replicates.

### Bacterial isolation from mammalian cells and bacterial metabolite extraction

When sampling from infection experiments for metabolite analysis, we seeded RAW264.7 or HeLa cells in T-75 flasks (Greiner). The gentamicin protection assay was performed as described before, with the addition of either unlabeled mannitol or U-^13^C mannitol at 0 hpi (when adding the low gentamicin concentration). At the sampling time point, if applicable, samples of the medium were taken, spun down once at 8,000 g for 5 min to remove potential cells, and 100 μL of the supernatant was added to 400 μL of an ice-cold 1:1 mixture of MeOH:ACN containing internal standard. The mixture was quickly vortexed and frozen at −80°C.

The infected cells were washed once with 10 mL of warm 0.9% NaCl and lysed using 3 mL ice-cold 0.05% Triton X-100 in NaCl on ice. The following steps were performed in a cold room on ice and samples were only processed in pairs or triplets to reduce processing time for each sample. The samples were incubated in the lysis solution for 5 min, scraped from the flask, and the lysate was filtered through a 5 μm cellulose nitrate filter (Whatman, Puradisc, WHA10462000). For HeLa cell samples (S3B Fig), the filtrates from 3 T-75 flasks were pooled for better coverage. The filtrate was then washed once with ice-cold NaCl in a pre-cooled centrifuge (10,000 g for 5 min at 1°C), and metabolites were extracted by resuspension in 100 μL of an ice-cold MeOH:ACN:H_2_O (40:40:20) mixture containing internal standard. The samples were immediately frozen at −80°C. This whole procedure took approximately 20 min.

Metabolites were extracted from the collected samples by subjecting them to 6 freeze–thaw cycles alternating between liquid nitrogen and shaking at 10°C. The cell debris was removed by centrifugation at 17,900 g for 10 min at 1°C, and the metabolite-containing supernatant was kept at −80°C until the analysis. To quantify absolute metabolite levels in the cell culture medium (S2I Fig), we prepared standard curves (2-fold dilutions) from fresh DMEM medium (1 g/L glucose, 0.11 g/L sodium pyruvate) which already contained most metabolites of interest at defined concentrations and supplemented it with additional compounds (mannitol, lactate, itaconate). All standard curves showed a linear or double-linear behavior in the sampled region. To obtain absolute concentrations from peak areas, we used the Matlab function *interp1()* for linear inter- and extrapolation.

### Preparation of bacterial in vitro samples for metabolomics

*S*Tm 14028S wt was diluted to OD 0.0003 from o/n cultures in M9 medium (1× M9 salts, 2 mM MgSO_4_, 0.1 mM CaCl_2_, 1 g/L amicase, and 4 g/L glucose or mannitol) and grown in M9 medium until approximately OD 0.15 in quadruplicates. Cells in volumes equivalent to OD 2 (i.e., 13.3 mL for OD 0.15) were spun down once at 4,000 g for 5 min, washed once with PBS, and metabolites were extracted by resuspension in 1 mL of an ice-cold MeOH:ACN:H_2_O (40:40:20) mixture containing internal standard (MSK-A2-1.2, Cambridge Isotope Laboratories). The samples were immediately frozen at −80°C and processed for metabolomics analysis as described above.

For the comparison of the fast and standard isolation protocol (S2E–S2G Fig), *S*Tm was grown in MOPS medium with 0.1% amicase and 0.4% mannitol (of which 50% was uniformly ^13^C-labeled), until an OD of approximately 0.2. For the fast isolation protocol, 5 mL of bacterial culture was filtered on a 0.45 μm polyvinylidene difluoride (PVDF) membrane (Merck Millipore) on a vacuum filtering flask, washed once with 5 mL warm PBS, and the filter was immediately placed into 1 mL ice-cold extraction solution (MeOH:ACN:H_2_O (40:40:20) mixture containing internal standard). For the standard isolation protocol, 10 mL of the same bacterial culture was pushed through a 5 μm cellulose nitrate filter (Whatman, Puradisc, WHA10462000) using a 50 mL syringe. The filtrate was spun down (4,000 g for 5 min at 1°C) and washed once with ice-cold PBS in a pre-cooled centrifuge (10,000 g for 5 min at 1°C), and metabolites were extracted by resuspension in a 1 mL extraction solution. All samples were immediately frozen at −80°C and processed further as described above. Experiments were performed in triplicates.

### LC–MS measurement

Most samples and standards were analyzed on a Vanquish UHPLC system coupled to a Q Exactive Plus HRMS (Thermo Fisher Scientific, Massachusetts, United States of America); samples on the effect of mannitol on bacterial and host cell physiology and the effect of the isolation protocol (“control samples,” S1G, S1I, S1J and S2E Figs) were analyzed on a Vanquish UHPLC system coupled to an Orbitrap Exploris 240 high-resolution mass spectrometer (Thermo Scientific, Massachusetts, USA) in negative ESI (electrospray ionization) mode. For most samples, the separation of metabolites was carried out on an XBridge BEH Amide column XP (100 × 2.1 mm; 2.5 μm) at a flow rate of 0.3 mL/min, maintained at 40°C. The mobile phase consisted of solvent A (7.5 mM ammonium acetate with 0.05% ammonium hydroxide) and solvent B (acetonitrile). A 16-min chromatographic run comprised a linear gradient from 2 to 12 min starting at 85% of solvent B and ending at 10%, followed by a hold from 12 to 14 min and a linear gradient to return to the initial conditions at 14.1 min. Analytes were recorded via a full scan with a mass resolving power of 70,000 over a mass range from 60 to 900 m/z, an AGC target of 10^6^ ions, and a maximum IT of 100 ms. Ion source parameters were set to the following values: spray voltage −3,500 V, sheath gas: 30 psi, auxiliary gas: 5 psi, S-Lens 65 eV, capillary temperature: 320°C, vaporizer temperature: 280°C. The column temperature was maintained at 40°C, the autosampler was set to 6°C and the sample injection volume was 3 μL for medium samples and 20 μL for cell samples. The instrument was calibrated before every analysis using a calibration solution (Thermo Fisher Scientific, #88340).

For the control samples (S1G, S1I, S1J and S2E Figs), chromatographic separation was carried out on an Atlantis Premier BEH Z-HILIC column (Waters, Massachusetts, USA; 2.1 mm × 100 mm, 1.7 μm) at a flow rate of 0.26 mL/min. The mobile phase consisted of H_2_O (mobile phase A) and ACN:H_2_O (9:1; mobile phase B), which were modified with a total buffer concentration of 10 mM ammonium acetate, respectively. The aqueous portion of each mobile phase was adjusted to pH 9.0 via the addition of ammonium hydroxide. The following gradient (20 min total run time including re-equilibration) was applied (min/%B): 0/100, 2/100, 15/50, 16/50, 16.5/100, 20/100. The column temperature was maintained at 40°C, the autosampler was set to 4°C and the sample injection volume was 7 μL. Analytes were recorded via a full scan with a mass resolving power of 180,000 over a mass range from 70 to 800 m/z (scan time: 100 ms, RF lens: 70%). Ion source parameters were set to the following values: spray voltage: −3,500 V, sheath gas: 30 psi, auxiliary gas: 5 psi, sweep gas: 0 psi, ion transfer tube temperature: 350°C, vaporizer temperature: 300°C.

Before sample analysis, metabolite standards were injected (standards listed under chemicals): we either injected them separately in extraction buffer or mixed them in spectrally non-overlapping groups based on their molecular weights into quality control (QC) samples. QC samples were prepared separately for the medium and cells by mixing equal volumes of their individual samples. This analysis of standards on the LC–MS system allowed us to determine retention times and peak shapes for all metabolites of interest.

Experimental samples were measured in a randomized manner. Pooled QC samples were prepared by mixing equal aliquots from each processed sample. Multiple QCs were injected at the beginning of the analysis to equilibrate the LC–MS system. A QC sample was analyzed after every approximately fifth experimental sample to monitor instrument performance throughout the analytical sequence. For the determination of background signals and subsequent background subtraction, an additional processed blank sample was recorded. Internal standards were used to detect procedural errors, not for data normalization.

### Analysis of metabolomics data

Peak areas of the deprotonated [M-H]^−^ metabolite ions and of all isotopologues (masses determined using Scientific Instrument Services isotope distribution calculator) for each metabolite were quantified on the smoothed extracted ion chromatograms (15 smoothing points) using the XCalibur Quan Browser software (Thermo Fisher Scientific, Version 4.1.31.9) with a mass tolerance of 7 to 10 ppm. Areas of non-detected peaks were set to 0, except stated otherwise.

All subsequent data analysis was done using Matlab. Based on the comparison between our standard and a faster isolation protocol, we considered the following metabolites as unreliable for the quantification of MIDs from intracellular *S*Tm. This selection was based on direct measurements or on close proximity to an unreliable metabolite in the metabolic network (although the metabolite could not be quantified in this specific experiment): cis-aconitate, citrate, α-ketoglutarate, glutamate, glutamine, serine, pyruvate, lactate, alanine, fumarate, aspartate (S1 Table). In addition, to identify real isotopic peaks—as opposed to contaminations or interference from other metabolites—we directly compared the same condition treated with ^12^C mannitol (unlabeled) and U-^13^C mannitol. As we generally had low signal intensities, we used the ^12^C samples to select metabolites for which we could make reliable statements about the quantitative fractional ^13^C enrichment in the ^13^C samples. This selection was done based on the detection of the naturally labeled M+1 isotope, and generally, only metabolites were selected for which the M+1 fractional enrichment did not deviate by more than 50% from the theoretically calculated M+1 enrichment (based on the natural ^13^C isotope abundance of 1.07% [77]), except for mannitol 1P and glucose 6P/fructose 6P in Fig 3, which deviated by 79% and 57%, respectively, for the low glucose data and mannitol 1P which deviated by 59% for the high glucose data (S4 Data). We chose this relatively high deviation of 50%, as orbitraps are already known to systematically underestimate heavier natural isotopes, up to 20% for the M+1 isotope [78]. In general, we expect higher labeling ratios in our data to be more accurate than smaller ones: as most labeled isotopologues showed higher labeling ratios than the naturally occurring ^13^C, we expect our data to be more accurate than the 50% cutoff. This strict cut-off led to a slightly different set of metabolites that could be quantified in each experiment. In general, the reproducibility and precision between replicates were very high (see S3A Fig), and we, therefore, decided to measure only 2 to 3 replicates per condition. Note that we only quantified labeling ratios from isolated bacteria, which are more stable than absolute metabolite levels [79] (and S2E Fig). We corrected all data for the ^13^C natural isotope and ^12^C impurity in the fully ^13^C-labeled mannitol using IsoCor [80].

To obtain the fractional ^13^C enrichment, we summed the peak areas from all reliably detected isotopes for one metabolite and divided the peak area of each isotope by this sum. Averages and standard deviations were determined from replicates using the Matlab functions *mean()*, and *std()*. To determine the total fractional ^13^C enrichment (TFLE) for one metabolite, we summed the percentages starting from M+1 (= 1 – M+0).

### Metabolic modeling and computational flux inference

A metabolic network model of central carbon metabolism comprising the Embden–Meyerhof pathway, the PPP, the EDP, the TCA cycle, anaplerosis, nucleotide biosynthesis, NADH biosynthesis, UDP-N-acetylglucosamine biosynthesis, and amino acids synthesized from these central metabolic pathways were formulated based on reactions taken from KEGG (https://www.genome.jp/kegg) for *Salmonella enterica* subsp. Typhimurium 14028S and equipped with carbon atom transitions acquired from BioCyc (https://biocyc.org) (S4 Fig). Unless thermodynamic evidence was available, all reactions were considered reversible, i.e., to carry a forward and a backward flux, in particular, the reactions of the reductive part of the PPP. The remaining (unidirectional) reactions, in particular, the uptake steps, are characterized by a single flux parameter. Some reactions catalyzed by more than one enzyme were formulated as a single reaction step. For example, the unidirectional phosphofructokinase (*pkfA/pfkB*) and the opposing unidirectional fructose-1,6-bisphosphatase (*fbp*) reactions were lumped into the bidirectional pseudo-reaction *pkfA/B_fbp*. Uptake reactions were specified for all potential major carbon substrates (mannitol, glucose/glucose 6P, gluconate, N-acetyl-glucosamine, ribose, mannose 6P, glycerol) that are accessible to *S*Tm [7]. Additionally, we allowed the uptake of amino acids, as well as nucleotides (adenine, guanine, cytosine, and uracil). For some metabolites (e.g., glycine or other riboses), no explicit uptakes were formulated, as these would be indistinguishable from neighboring uptakes (which is to be kept in mind when interpreting their contributions). Intracellular bacterial growth was modeled by precursor drain fluxes (green in S4 Fig), with values representing their share in the overall biomass composition [81]. Then, from the central carbon metabolism model, a focused model was formulated by pruning TCA and anaplerotic pathways. That reduced the number of amino acids potentially taken up to alanine, tyrosine, phenylalanine, tryptophan, histidine, and serine (cyan in S4 Fig).

Unless otherwise stated, reaction fluxes were formulated in the net-exchange flux coordinate system [53]. Net fluxes are expressed relative to the growth rate μ. Except for CO_2_ production, net fluxes were constrained between −15 and 15 times μ, whereas exchange fluxes were constrained by 30 times μ. From simulations with the large model, calibrated with the labeling data, we derived further constraints on net fluxes, which were applied in the focused model. Specifically, these were upper bounds for the summed net uptake of and outflux from pyruvate (1.0 · μ), and lower and upper bounds for the *pgk/gpm/eno* (PGP → PEP) flux, i.e., 0.4 · μ ≤ *pgk/gpm/eno* ≤ 1.3 · μ. Furthermore, fluxes from pyruvate towards the TCA and the anaplerotic pathways as well as potential backward (gluconeogenetic) fluxes in the focused model were constrained by values obtained from full model simulations.

The purity of mannitol was set to the manufacturer-specified value of 99 atom%. Naturally occurring heavy ^13^C atoms from naturally labeled carbons of the tracer are considered by the simulation model, as well as ^12^C atoms from the impurity of labeled carbon atoms. For both configurations, i.e., low/high glucose concentrations, measured isotopologues were corrected for the natural abundance of other heavy isotopes [80] and incorporated into the model if found reliable (S1 Table, S2E Fig, and Methods), resulting in a total of 127 single measurements for 12 metabolites for each glucose concentration. Since glucose 6P and fructose 6P could not be separated, a mixed pool (H6P) was introduced to model their sum measurement. The atom transition model was formulated in the standardized FluxML document format [82]. Labeling data for the two glucose concentrations were included as two different configurations (S1 File).

BMA using a tailored Markov chain Monte Carlo (MCMC) approach [55] and the high-performance ^13^C MFA simulator 13CFLUX2 [83] was employed, delivering joint posterior probability distribution for the net fluxes. From the marginal posterior distributions 95% credible intervals were extracted (S5A Fig), as well as the probability for the intracellular reactions to operate in forward and backward directions simultaneously (S2 Table). For the latter, the probabilities for forward (backward) fluxes were calculated as the proportion of samples for which the net flux was >0 (<0) plus the proportion of samples for which the net flux was <0 (>0) and the exchange flux was non-zero.

For MCMC, 10 Markov chains were run. For all net fluxes, a potential scale reduction factor (PSRF) below 1.1 (S3 Table) indicated proper convergence [84] after burn-in (6,000,000 samples) and thinning (discarding all but every 2,500 samples from a total of 10,000,000 samples), resulting in 10 times 4,000 samples. To assess the quality of the samples, mixing plots for the net fluxes (S8 Fig) and of the model space (S9 Fig) were checked for proper mixing. Finally, predictive posterior checks were performed to assure that the model used for inference is consistent with the labeling data (S10 Fig).

### SDS-PAGE and immunoblot

To determine the contribution of bacterial material to the total cell material during an infection (S2A Fig), uninfected host RAW264.7 cells, and host cells infected with a replication-deficient mutant (Δ*ssaV*), or the wt were separated at 20 hpi by SDS-PAGE alongside serially diluted *S*Tm grown in monoculture (1:1, 1:10, 1:100, 1:300, 1:900). Sample preparation and immunoblot using anti-RecA were performed as described below. To determine the contamination of host material during our isolation protocol (S2B and S2C Fig) (as described above but using cOmplete protease inhibitor cocktail (Roche, 11697498001) in the lysis buffer Triton X-100 in PBS), samples were taken at different steps of the bacterial isolation protocol: before filtration through the 5 μm filter, after filtration, before, and after the concentration step. The samples were added to Laemmli buffer loading dye, heated to 95°C for 5 min, and centrifuged. Samples were then separated by SDS-PAGE and followed by immunoblot transfer to a 0.45 μm PVDF membrane (Millipore, Immobilin-P, no. IPVH00010). Membranes were blocked with 5% skim milk in Tris-buffered saline (TBS-T) for 1 h and probed with the following antibodies by incubation at 4°C overnight: RecA (Abcam ab63797), 1:5,000; GAPDH (Cell Signaling #8884), 1:10,000; VDAC1 (Cell Signaling #4866), 1:1,000; histone H3 (Cell Signaling #4499), 1:1,000; Calreticulin (Cell Signaling #12238), 1:1,000. The secondary antibody (anti-rabbit) was used at 1:10,000 and incubated with washed membranes for 1 h at room temperature. After washing, a chemiluminescence substrate (Thermo Fisher Scientific #32109) was used for signal development and detected using a digital developing machine (ChemiDoc touch imaging system). Protein quantification by densitometry was performed using ImageJ version 1.52i.

### Isolation and hydrolysis of RNA from bacteria

After the filtration of bacteria from host cell lysate (described above), bacterial total RNA was isolated using the RNeasy Protect Bacteria Mini Kit (Qiagen). Bacterial RNA was then digested by the nucleoside digestion mix (NEB, M0649S), nucleosides were extracted by the addition of 80 μL MeOH:ACN mixture (1:1) to 20 μL nucleoside mix, and the samples were analyzed using LC–MS as described above. Nucleobases (without the ribose unit) were detectable and probably generated by in-source fragmentation in the mass spectrometer, as their retention times matched with their corresponding nucleosides.

## Supporting information

S1 Fig Mannitol uptake is repressed in vitro, but mannitol reaches *S*Tm inside host cells without affecting the host or bacterial metabolism. (A) ^13^C-labeled fractions of hexose-phosphates and alanine from HeLa cells, determined 48 h after the addition of U-^13^C mannitol (mtl), or U-^13^C glucose (glc) into the glc-containing cell culture medium (DMEM with 1 g/L glc, Methods). Graphs show averages from 3 biological replicates. (B) Growth of *S*Tm wt, and ∆*mtlD*, in MOPS medium with amino acids (Methods) and with combinations of glucose 6P (glc6P), and mtl, as the main carbon source. Bars depict the average of 4 technical replicates from 1 biological replicate. (C) In vitro growth curves of wt, ∆*mtlA*, and ∆*mtlD* under repressive and non-repressive conditions, in MOPS medium with different carbon sources (combinations of glc, mtl, glycerol (glyc), and glc6P). Curves are representative of at least 2 technical replicates and 2 independent experiments (1 independent experiment for plots with glc6P). (D) The same as (C) but with wt and a knockout mutant of glycerol-3P dehydrogenase (∆*glpD)* which accumulates toxic glycerol phosphate [42]. While mtl represses the uptake of glycerol, the non-repressive carbon source pyruvate (pyr) does not, thereby reducing the growth rate of ∆*glpD*. Curves are representative of 2 biological replicates. (E) *S*Tm ∆*mtlD* isolated from HeLa cells after a gentamicin protection assay, supplemented with or without mtl during the infection and spotted on an LB agar plate. The image is representative of biological triplicates. (F) Exponential growth rates of wt in M9 medium with and without amino acids (AA; Methods), compared between glc and mtl as the carbon source. (G) Volcano plot of 67 relative metabolite levels of wt grown in M9 medium with mtl versus glc as the carbon source. Data are from 4 biological replicates (S2 Data). Dashed lines show a 2-fold increase/decrease. (H) Fold change in CFUs of *S*Tm in the *glpD* mutant compared to the wt at 12 hpi in RAW264.7 cells with or without the addition of mtl or glyc. Bars are averages of 6 biological replicates. *p*-values are highly significant (8.3·10^-7^ and 6.5·10^-4^) from a two-sample *t* test. (I) Volcano plot of 69 relative metabolite levels quantified from RAW264.7 cells 12 h after or without the addition of 4g/L mtl. (J) Same as (I) but for 67 metabolite levels in HeLa cells. Data in (I) and (J) are from 6 biological replicates. Dashed lines show a 2-fold increase/decrease (S3 Data). The data underlying this figure can be found in S1 Data.
(TIF)

S2 Fig Bacterial isolation and optimal sampling time point allow robust quantification of metabolites. (A) Uninfected host RAW264.7 cells (denoted by “-”), and host cells infected with a replication-deficient mutant (Δ*ssaV*), or the wt were separated at 20 hpi by SDS-PAGE alongside serially diluted *S*Tm grown in monoculture (dilutions denoted above blot). Immunoblot was performed using an antibody against the bacterial RecA protein (38 kDa) for the quantification of bacterial material among host material in the infection assay by densitometry (numbers (a.u.) on the blot) via a standard curve. The experiment was performed in biological duplicates (S1 Raw Image). (B) Enrichment/depletion after filtration, and washing/concentration applied to infected RAW264.7 cells. Fold protein levels relative to the level before the filtration are shown for bacteria (anti-RecA), host cytosolic proteins (anti-GAPDH), mitochondria (anti-VDAC1), histones from nuclei (anti-H3), and the endoplasmic reticulum (ER, anti-Calreticulin). Mitochondria and nuclei were removed by the filtering step, and solubilized ER and cytosolic proteins by centrifuging and washing the bacterial-containing flow-through. Bars are the averages of triplicates. Quantification by densitometry from immunoblots (Methods). (C) Same as in (B) but with the bacterial enrichment protocol applied to infected HeLa cells. Using the same protocol, bacterial enrichment from HeLa cells is less strong compared to RAW264.7 cells, reflecting physiological differences between the two different cell lines. Bars are the averages from biological duplicates. (D) Same data as in Fig 2D, but ratios of peak areas of control over the sample are separately depicted here for each metabolite. The solid, dashed, and dotted lines indicate the height at which the control reaches 100%, 50%, or 20%, respectively, of the peak area of the sample. Data are representative of 2 independent experiments in infected host RAW264.7 cells. (E) Total fractional labeling enrichment (TFLE, *n* = 38) from bacterial monocultures fed with 50% U-^13^C-labeled mannitol compared between the standard and the fast enrichment protocol. Green dots indicate metabolites with significantly different (adjusted *p*-value <0.01) TFLE between both conditions (“unreliable metabolites”). The dashed line indicates equal TFLEs from both protocols. Note that we considered pyruvate-derived metabolites (alanine, lactate) as unreliable, as we could not quantify them in this experiment. NADH is a false positive, as the ^13^C labeling pattern is highly similar between the standard and fast protocol (insert). Dots and lines are the averages and standard deviations, respectively, from biological triplicates. (F) MIDs for the fast and standard isolation protocol from bacterial monocultures for selected metabolites with significantly different TFLEs, but only quantitatively different MIDs. (G) Fumarate also showed qualitatively different MIDs between the fast and the standard protocol. Bars in (F, G) are averages of biological triplicates. (H) Replication dynamics of *S*Tm inside HeLa cells in a mtl-containing medium. Line is the average of biological triplicates. (I) Absolute metabolite concentrations in the cell culture medium during *S*Tm replication inside RAW264.7 and HeLa cells. Lines are the average of biological duplicates. The black line on the right side of each figure indicates the concentration range covered by the standard curve. (J) Fractional ^13^C labeling enrichment for 31 metabolites (their 244 isotopologues are compared) from bacteria isolated from RAW264.7 cells compared between 12 and 16 hpi (r is the Pearson correlation). Dots are averages from biological duplicates. The data underlying this figure can be found in S1 Data.
(TIF)

S3 Fig Replicate correlations and comparison of bacterial metabolite labeling from U-^13^C mannitol between two cell lines. (A) Pairwise replicate correlations (triplicates) from the fractional ^13^C enrichment of *n* = 183 isotopologues in 24 metabolites from RAW264.7 cells from the same experiment as shown in Fig 3A. The Pearson correlation coefficient r is depicted. (B) Total fractional labeling enrichment of different bacterial metabolites (*n* = 17) isolated from and compared between RAW264.7 and HeLa cells 12 hpi with MOI 100. Bars show averages of 3 (RAW264.7) and 2 (HeLa) biological replicates. Bars for RAW264.7 cells are the same as in Fig 3A. Metabolites quantitatively affected by the enrichment protocol (S2E Fig) are denoted with a *. The data underlying this figure can be found in S1 Data.
(TIF)

S4 Fig Graphical representation of the focused metabolic network model of *S*Tm. Network construction and visualization were performed using the network editor and visualization software Omix [85]. Small double-headed arrows indicate reaction reversibility. The model contains the following pathways: glycolysis (red), pentose phosphate pathway (PPP; orange), Entner–Doudoroff pathway (EDP; purple), biomass synthesis (bm; green), further uptake reactions (cyan), amino acid biosynthesis (blue), nucleotide biosynthesis (brown), NAD synthesis (violet), and biosynthesis of UDP N-acetylglucosamine (in yellow). Uptake was also allowed for all relevant included amino acids and nucleotides. Metabolite names not mentioned before are: Glcn6P: 6-phosphogluconate, Man6P: mannose 6P, PGP: phosphoglycerate, AcCoA: Acetyl-CoA, GlcNAc: N-acetylglucosamine, GlcNAcP: N-acetylglucosamine-phosphate, UDPGlcNAc: UDP N-acetylglucosamine, ORO: orotate, NCLA: N-carbamoyl-L-aspartate, FGAM: 5-phosphoribosyl-N-formylglycineamidine, GAR: 5-phosphoribosyl-glycineamide, AICAR: 5′-phosphoribosyl-5-amino-4-imidazole carboxamide, FAICAR: 5-formamido-1-(5-phospho-D-ribosyl)-imidazole-4-carboxamide, PRA: 1-(5-phospho-β-D-ribosyl)-AMP, IAP: 3-(imidazol-4-yl)-2-oxopropyl phosphate, KIV: 2-keto-isovalerate, THF: tetrahydrofolate, QUI: quinolinate. Amino acids and nucleotides in standard 3-letter abbreviations.
(TIF)

S5 Fig Credible intervals for inferred net fluxes in the low glucose condition and a condition with more glucose (high glucose) in the host medium. (A) The 95% credible intervals for net fluxes of major central carbon metabolism pathways (glycolysis, EDP, PPP, nutrient uptake, and nucleotide synthesis) for the low glucose and high glucose condition. (B) Glucose (glc) concentrations in our standard condition (low glc) and in a condition with a higher glc concentration in the mammalian growth medium (high glc). (C) Unlabeled fractions of 22 bacterial metabolites compared in the low and high glc conditions (S4 Data). The data underlying this figure can be found in S1 Data.
(TIF)

S6 Fig Mass isotopomer distributions from isolated bacterial RNA after infection. (A, B) The fractional ^13^C enrichment of adenosine and uridine (with the ribose units) in bacterial RNA isolated 20 hpi with MOI 100 from RAW264.7 macrophages. Bars are the averages of biological duplicates. The data underlying this figure can be found in S1 Data.
(TIF)

S7 Fig Mass isotopomer distributions for succinate, TFLEs compared between wt and *ppc* knockout mutant and growth of the mutant in vitro. (A) Mass isotopomer distributions for succinate in the wt. Bars are averages from biological triplicates. (B) TFLE correlation compared between the wt and *ppc* mutant based on 32 metabolites. (C) Mass isotopomer distributions for succinate in the *ppc* knockout mutant. Bars are averages from biological triplicates. (D) In vitro growth of *S*Tm wt and ∆*ppc* in rich LB and minimal M9 glucose medium. The data underlying this figure can be found in S1 Data.
(TIF)

S8 Fig Mixing plots of the free net fluxes (1 representative replicate out of 10). The MCMC algorithm is run for 1.6·10^7^ iterations using 10 independent chains. The first 6·10^6^ samples were treated as burn-in and were, therefore, discarded. Of the remaining 10^7^ samples, each 2,500th sample was saved (thinning), yielding 4,000 samples per MCMC replicate. The trace plots indicate the proper mixing of the Markov chain in the flux space. (A) Low glucose concentration, (B) high glucose concentration.
(TIF)

S9 Fig Mixing plots in model space (varying in reaction bidirectionalities, all replicates). To certify that the replicates did not get stuck in different regions of model space, all replicates are shown. Each subplot represents 1 independent MCMC run. The plots show that the sampler mixes well in the model space and that the mixing is very reproducible for all 10 replicate chains. (A) Low glucose concentration, (B) high glucose concentration.
(TIF)

S10 Fig Posterior predictive checks. Simulated mass isotopomer distributions (colored bars depict the 95% interval of the simulated measurements), sampled according to the flux posterior distributions obtained from BMA-based ^13^C MFA inference compared to measured values (black crosses are means with lines as standard deviations). The plots show that the model fits the data well in both cases, the low glucose concentration (blue) and the high glucose concentration (orange). The data underlying this figure can be found in S1 Data.
(TIF)

S1 Raw Image Original high-resolution images of the western blot membrane depicted in S2 Fig, in 2 replicates. The data underlying these images can be found in S1 Data.
(PDF)

S1 File Metabolic model including atom transitions, constraints, and labeling measurements used for the analyses in the standardized FluxML document format. (FML)

S1 Data Data underlying all figures and supplementary figures. (XLSX)

S2 Data Metabolite levels (a.u.) quantified from *S*Tm monocultures growing in MOPS glucose or mannitol medium. (XLS)

S3 Data Metabolite levels (a.u.) quantified from host cells (RAW264.7, HeLa) -/+ the addition of mannitol. (XLS)

S4 Data Fractional ^13^C enrichment of *S*Tm isolated from RAW264.7 cells for low and high glucose medium. (XLS)

S1 Table Metabolites for which MIDs could be quantified, were reliable using our standard isolation protocol (S2E Fig), were used to determine pathway activity (Figs 3A and S3B), were used for isotope tracing (Fig 6), or were included in the metabolic model (Figs 4 and S4); n.d. stands for not determined. (XLSX)

S2 Table Fraction of forward and backward fluxes being active. For each potentially bidirectional reaction, it is counted how often the forward and backward flux samples had non-zero values in the MCMC runs. If the value is above 95%/below 5% the direction is assumed to be active (denoted by “+”)/inactive (denoted by “-”), otherwise, the data is inconclusive (denoted by “?”). The principal direction is inferred from 95% net flux credible intervals.
(XLSX)

S3 Table PSRF from the Markov chains. A value below 1.1 indicates proper convergence.
(XLSX)

## Abbreviations

a.u.: arbitrary unit
AA: amino acids
BMA: Bayesian model averaging
CFU: colony-forming unit
EDP: Entner–Doudoroff pathway
ER: Endoplasmic Reticulum
ESI: electrospray ionization
glc: glucose
hpi: hours post-infection
LC-MS: liquid chromatography-mass spectrometry
LPS: lipopolysaccharide
MCMC: Markov chain Monte Carlo
MFA: metabolic flux analysis
MID: mass isotopomer distribution
MOI: multiplicity of infection
MS: mass spectrometry
mtl: mannitol
NMR: nuclear magnetic resonance
PEP: phosphoenolpyruvate
ppc: phosphoenolpyruvate carboxylase
PPP: pentose phosphate pathway
PSRF: potential scale reduction factor
PTS: phosphotransferase system
PVDF: polyvinylidene difluoride
QC: quality control
SCV: *Salmonella*-containing vacuole
*S*Tm: *Salmonella* Typhimurium
TCA: tricarboxylic acid
TFLE: total fractional labeling enrichment
U-^13^C: uniformly ^13^C-labeled
wt: wild type
