# Supplementary figures and images for: A pathogen-specific isotope tracing approach reveals metabolic activities and fluxes of intracellular Salmonella
Source: PLoS Biol. 2023 Aug 18;21(8):e3002198. doi: 10.1371/journal.pbio.3002198 (PMC10468081; doi:10.1371/journal.pbio.3002198)

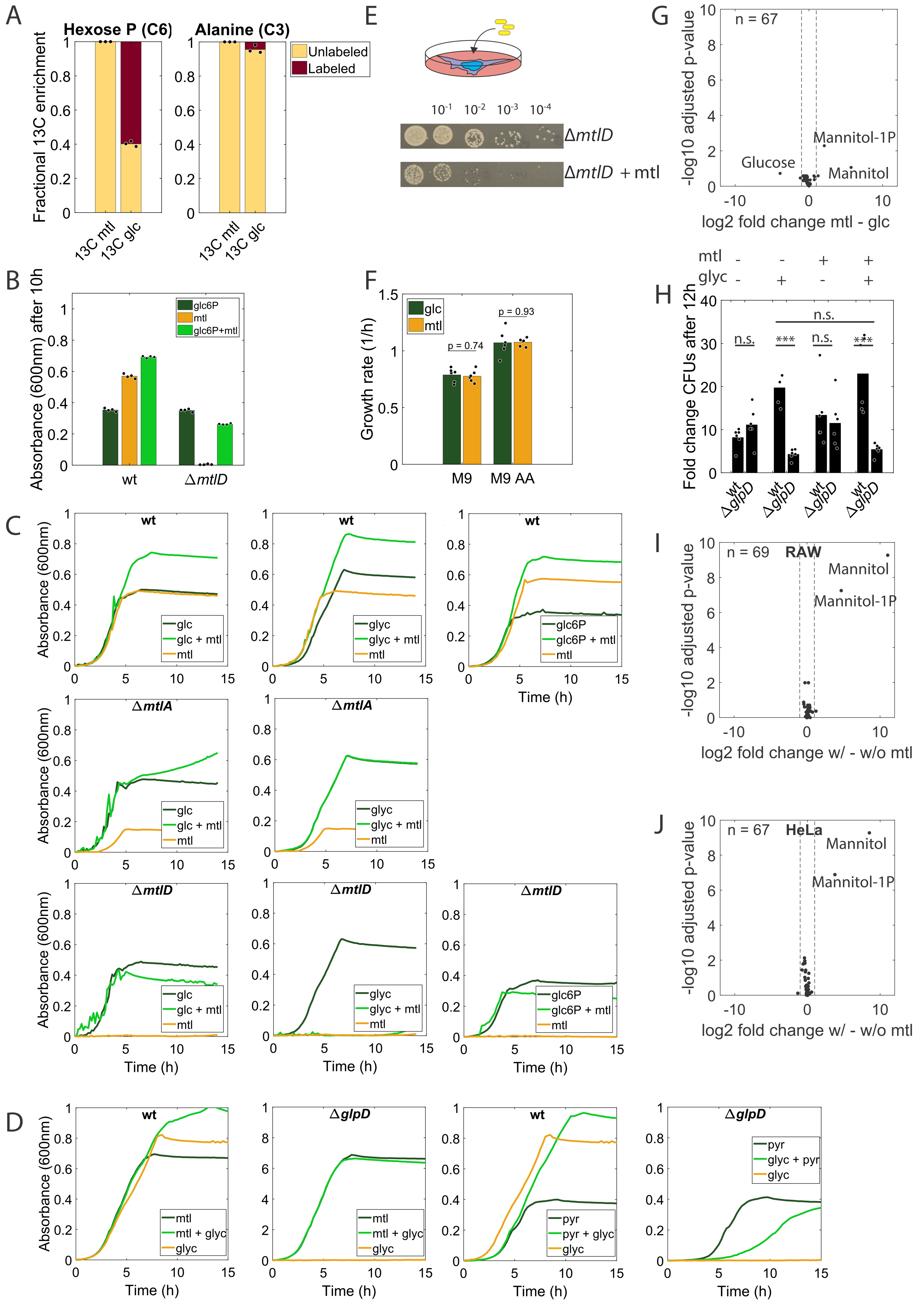

Supplement: S1 Fig — (A) 13C-labeled fractions of hexose-phosphates and alanine from HeLa cells, determined 48 h after the addition of U-13C mannitol (mtl), or U-13C glucose (glc) into the glc-containing cell culture medium (DMEM with 1 g/L glc, Methods). Graphs show averages from 3 biological replicates. (B) Growth of STm wt, and ∆mtlD, in MOPS medium with amino acids (Methods) and with combinations of glucose 6P (glc6P), and mtl, as the main carbon source. Bars depict the average of 4 technical replicates from 1 biological replicate. (C) In vitro growth curves of wt, ∆mtlA, and ∆mtlD under repressive and non-repressive conditions, in MOPS medium with different carbon sources (combinations of glc, mtl, glycerol (glyc), and glc6P). Curves are representative of at least 2 technical replicates and 2 independent experiments (1 independent experiment for plots with glc6P). (D) The same as (C) but with wt and a knockout mutant of glycerol-3P dehydrogenase (∆glpD) which accumulates toxic glycerol phosphate [42]. While mtl represses the uptake of glycerol, the non-repressive carbon source pyruvate (pyr) does not, thereby reducing the growth rate of ∆glpD. Curves are representative of 2 biological replicates. (E) STm ∆mtlD isolated from HeLa cells after a gentamicin protection assay, supplemented with or without mtl during the infection and spotted on an LB agar plate. The image is representative of biological triplicates. (F) Exponential growth rates of wt in M9 medium with and without amino acids (AA; Methods), compared between glc and mtl as the carbon source. (G) Volcano plot of 67 relative metabolite levels of wt grown in M9 medium with mtl versus glc as the carbon source. Data are from 4 biological replicates (S2 Data). Dashed lines show a 2-fold increase/decrease. (H) Fold change in CFUs of STm in the glpD mutant compared to the wt at 12 hpi in RAW264.7 cells with or without the addition of mtl or glyc. Bars are averages of 6 biological replicates. p-values are highly significant (8.3·10 [file pbio.3002198.s001.tif]

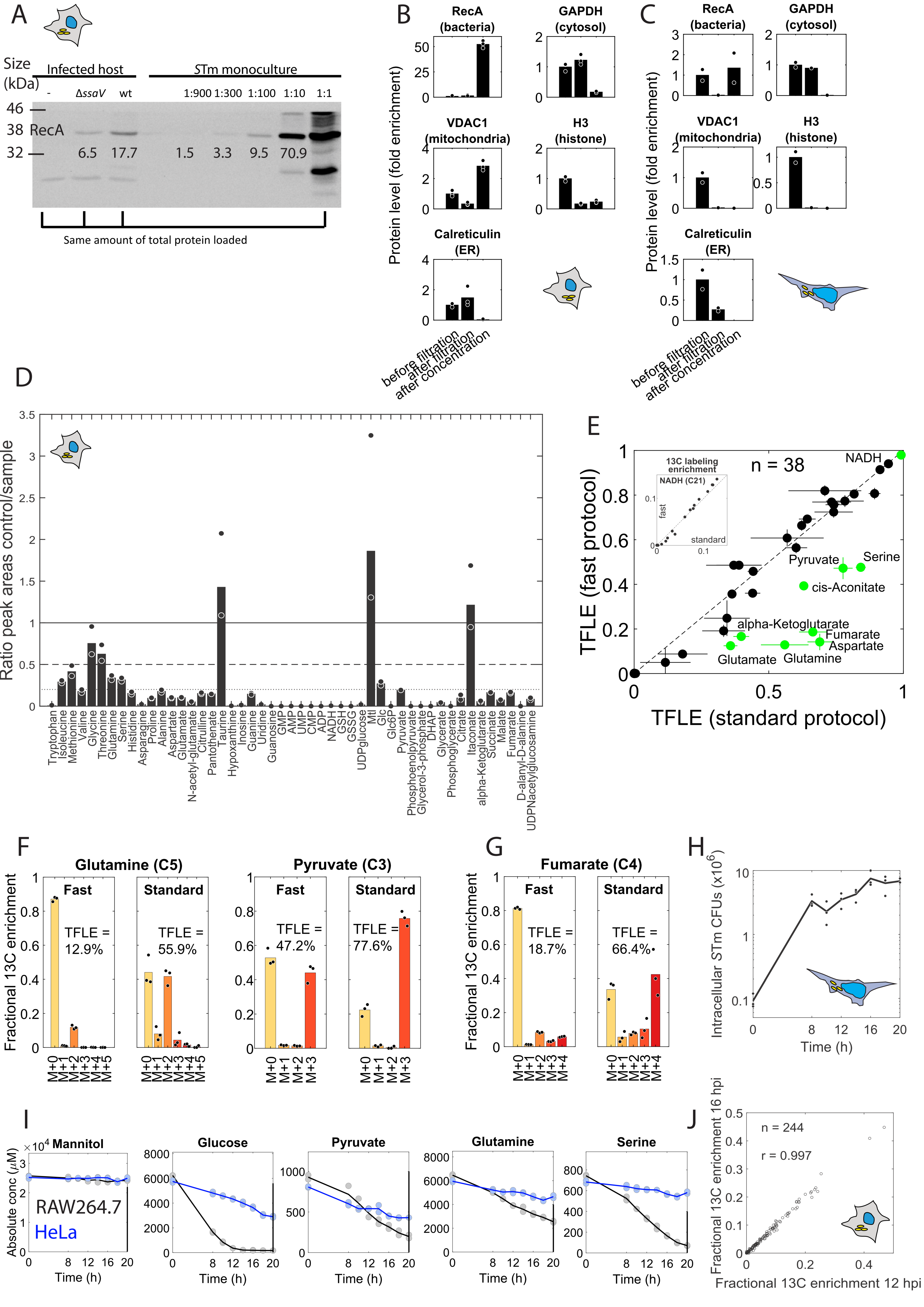

Supplement: S2 Fig — (A) Uninfected host RAW264.7 cells (denoted by “-”), and host cells infected with a replication-deficient mutant (ΔssaV), or the wt were separated at 20 hpi by SDS-PAGE alongside serially diluted STm grown in monoculture (dilutions denoted above blot). Immunoblot was performed using an antibody against the bacterial RecA protein (38 kDa) for the quantification of bacterial material among host material in the infection assay by densitometry (numbers (a.u.) on the blot) via a standard curve. The experiment was performed in biological duplicates (S1 Raw Image). (B) Enrichment/depletion after filtration, and washing/concentration applied to infected RAW264.7 cells. Fold protein levels relative to the level before the filtration are shown for bacteria (anti-RecA), host cytosolic proteins (anti-GAPDH), mitochondria (anti-VDAC1), histones from nuclei (anti-H3), and the endoplasmic reticulum (ER, anti-Calreticulin). Mitochondria and nuclei were removed by the filtering step, and solubilized ER and cytosolic proteins by centrifuging and washing the bacterial-containing flow-through. Bars are the averages of triplicates. Quantification by densitometry from immunoblots (Methods). (C) Same as in (B) but with the bacterial enrichment protocol applied to infected HeLa cells. Using the same protocol, bacterial enrichment from HeLa cells is less strong compared to RAW264.7 cells, reflecting physiological differences between the two different cell lines. Bars are the averages from biological duplicates. (D) Same data as in Fig 2D, but ratios of peak areas of control over the sample are separately depicted here for each metabolite. The solid, dashed, and dotted lines indicate the height at which the control reaches 100%, 50%, or 20%, respectively, of the peak area of the sample. Data are representative of 2 independent experiments in infected host RAW264.7 cells. (E) Total fractional labeling enrichment (TFLE, n = 38) from bacterial monocultures fed with 50% U-13C-labeled mannitol co [file pbio.3002198.s002.tif]

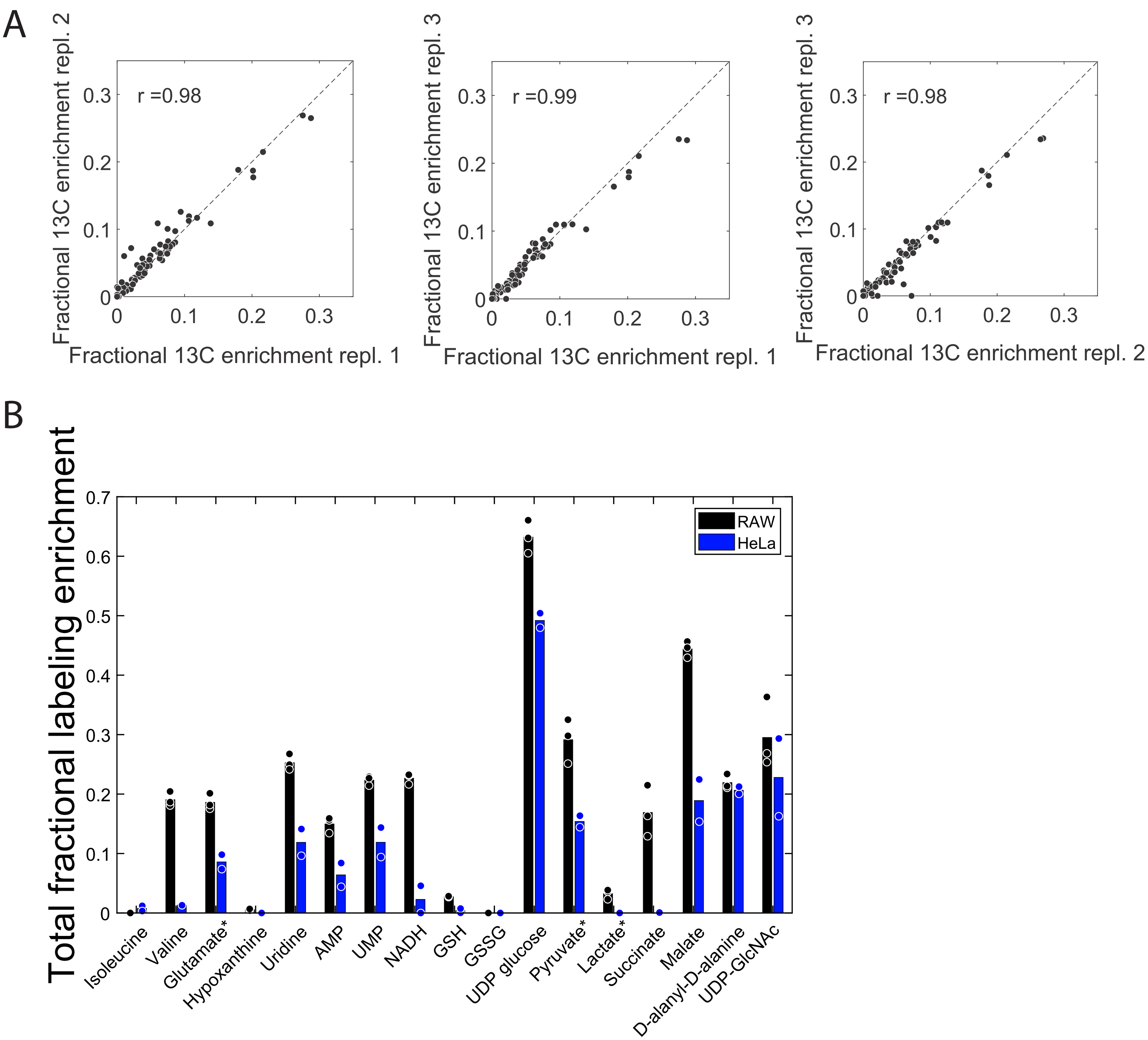

Supplement: S3 Fig — (A) Pairwise replicate correlations (triplicates) from the fractional 13C enrichment of n = 183 isotopologues in 24 metabolites from RAW264.7 cells from the same experiment as shown in Fig 3A. The Pearson correlation coefficient r is depicted. (B) Total fractional labeling enrichment of different bacterial metabolites (n = 17) isolated from and compared between RAW264.7 and HeLa cells 12 hpi with MOI 100. Bars show averages of 3 (RAW264.7) and 2 (HeLa) biological replicates. Bars for RAW264.7 cells are the same as in Fig 3A. Metabolites quantitatively affected by the enrichment protocol (S2E Fig) are denoted with a *. The data underlying this figure can be found in S1 Data. (TIF) [file pbio.3002198.s003.tif]

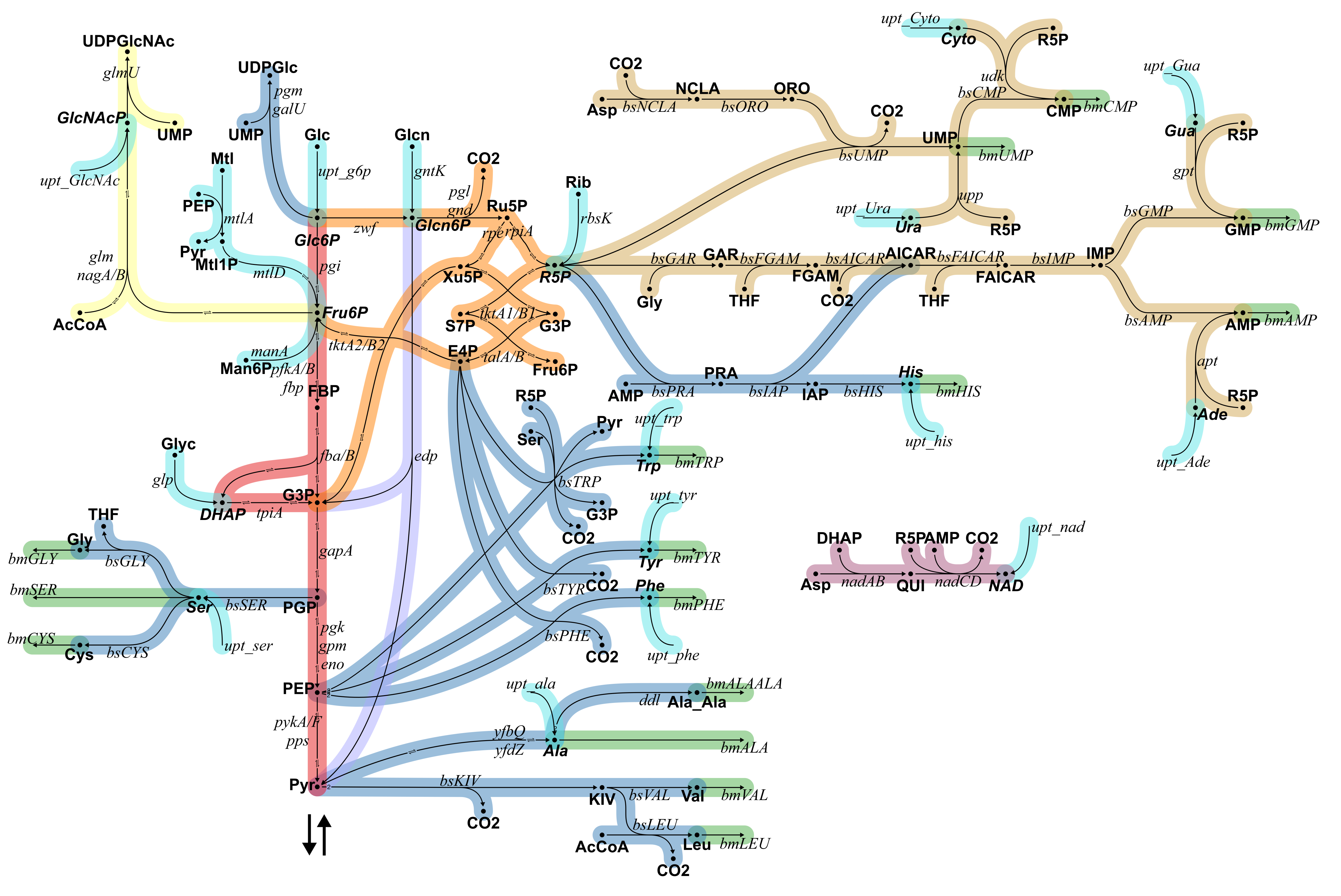

Supplement: S4 Fig — Network construction and visualization were performed using the network editor and visualization software Omix [85]. Small double-headed arrows indicate reaction reversibility. The model contains the following pathways: glycolysis (red), pentose phosphate pathway (PPP; orange), Entner–Doudoroff pathway (EDP; purple), biomass synthesis (bm; green), further uptake reactions (cyan), amino acid biosynthesis (blue), nucleotide biosynthesis (brown), NAD synthesis (violet), and biosynthesis of UDP N-acetylglucosamine (in yellow). Uptake was also allowed for all relevant included amino acids and nucleotides. Metabolite names not mentioned before are: Glcn6P: 6-phosphogluconate, Man6P: mannose 6P, PGP: phosphoglycerate, AcCoA: Acetyl-CoA, GlcNAc: N-acetylglucosamine, GlcNAcP: N-acetylglucosamine-phosphate, UDPGlcNAc: UDP N-acetylglucosamine, ORO: orotate, NCLA: N-carbamoyl-L-aspartate, FGAM: 5-phosphoribosyl-N-formylglycineamidine, GAR: 5-phosphoribosyl-glycineamide, AICAR: 5′-phosphoribosyl-5-amino-4-imidazole carboxamide, FAICAR: 5-formamido-1-(5-phospho-D-ribosyl)-imidazole-4-carboxamide, PRA: 1-(5-phospho-β-D-ribosyl)-AMP, IAP: 3-(imidazol-4-yl)-2-oxopropyl phosphate, KIV: 2-keto-isovalerate, THF: tetrahydrofolate, QUI: quinolinate. Amino acids and nucleotides in standard 3-letter abbreviations. (TIF) [file pbio.3002198.s004.tif]

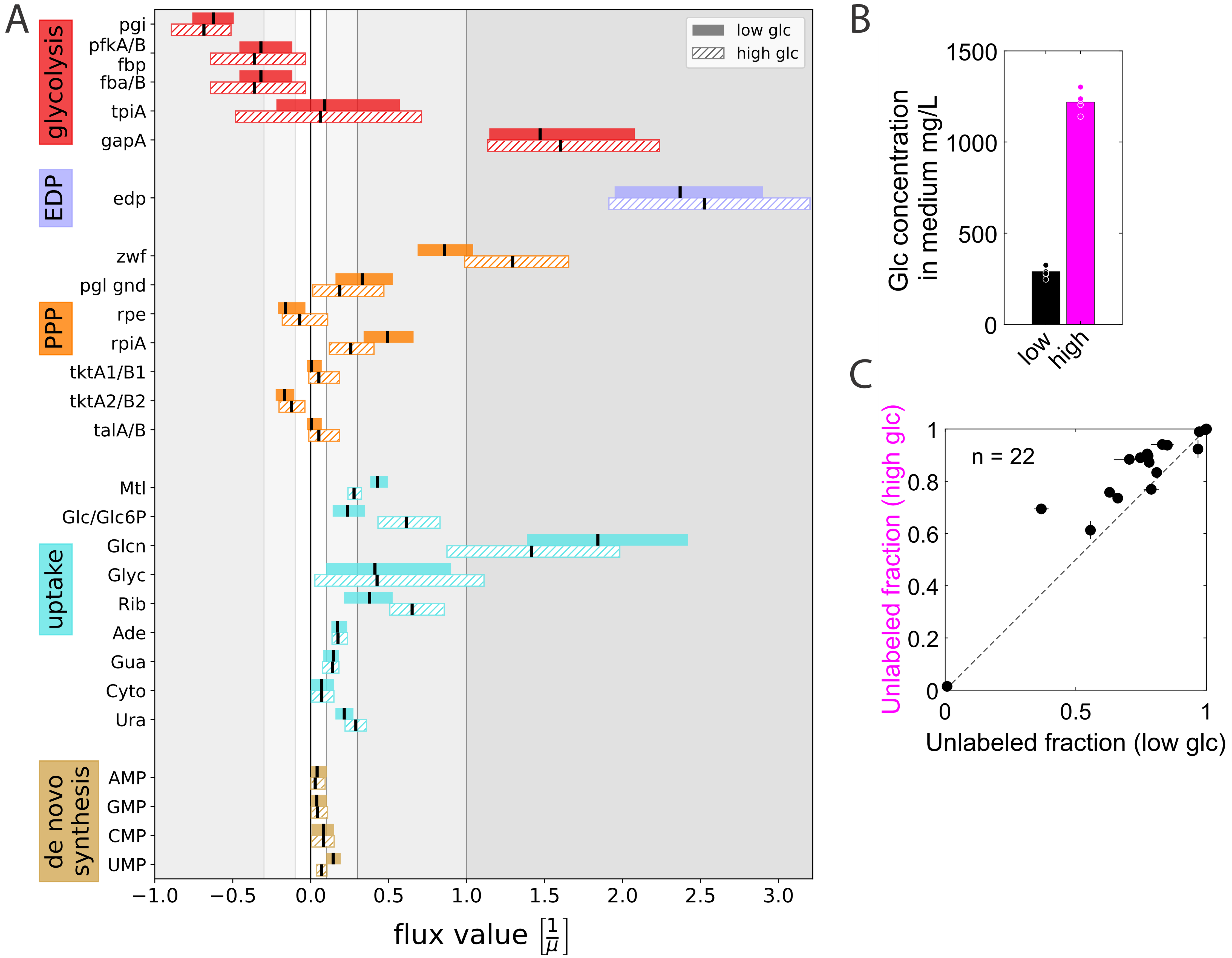

Supplement: S5 Fig — (A) The 95% credible intervals for net fluxes of major central carbon metabolism pathways (glycolysis, EDP, PPP, nutrient uptake, and nucleotide synthesis) for the low glucose and high glucose condition. (B) Glucose (glc) concentrations in our standard condition (low glc) and in a condition with a higher glc concentration in the mammalian growth medium (high glc). (C) Unlabeled fractions of 22 bacterial metabolites compared in the low and high glc conditions (S4 Data). The data underlying this figure can be found in S1 Data. (TIF) [file pbio.3002198.s005.tif]

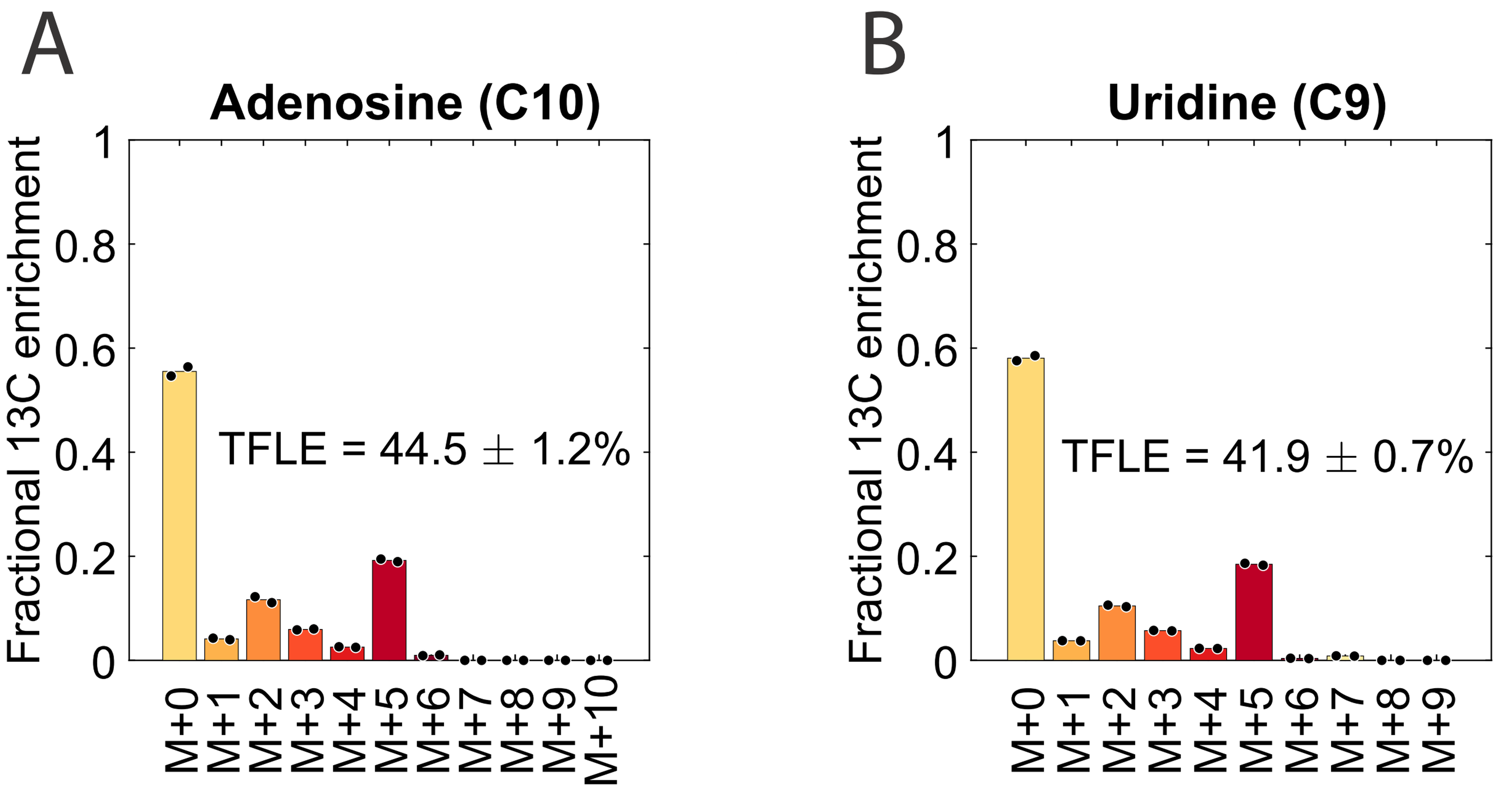

Supplement: S6 Fig — (A, B) The fractional 13C enrichment of adenosine and uridine (with the ribose units) in bacterial RNA isolated 20 hpi with MOI 100 from RAW264.7 macrophages. Bars are the averages of biological duplicates. The data underlying this figure can be found in S1 Data. (TIF) [file pbio.3002198.s006.tif]

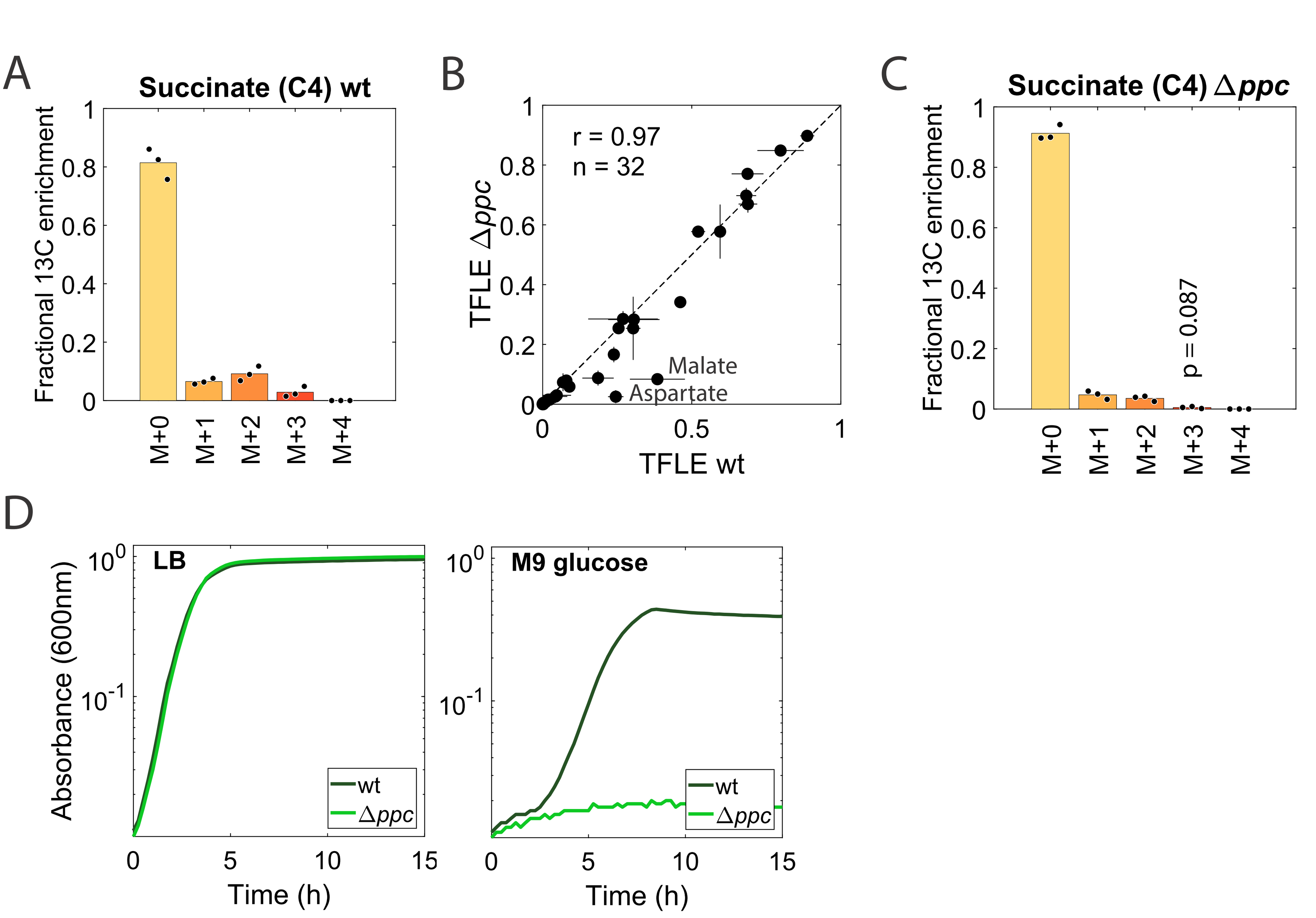

Supplement: S7 Fig — (A) Mass isotopomer distributions for succinate in the wt. Bars are averages from biological triplicates. (B) TFLE correlation compared between the wt and ppc mutant based on 32 metabolites. (C) Mass isotopomer distributions for succinate in the ppc knockout mutant. Bars are averages from biological triplicates. (D) In vitro growth of STm wt and ∆ppc in rich LB and minimal M9 glucose medium. The data underlying this figure can be found in S1 Data. (TIF) [file pbio.3002198.s007.tif]

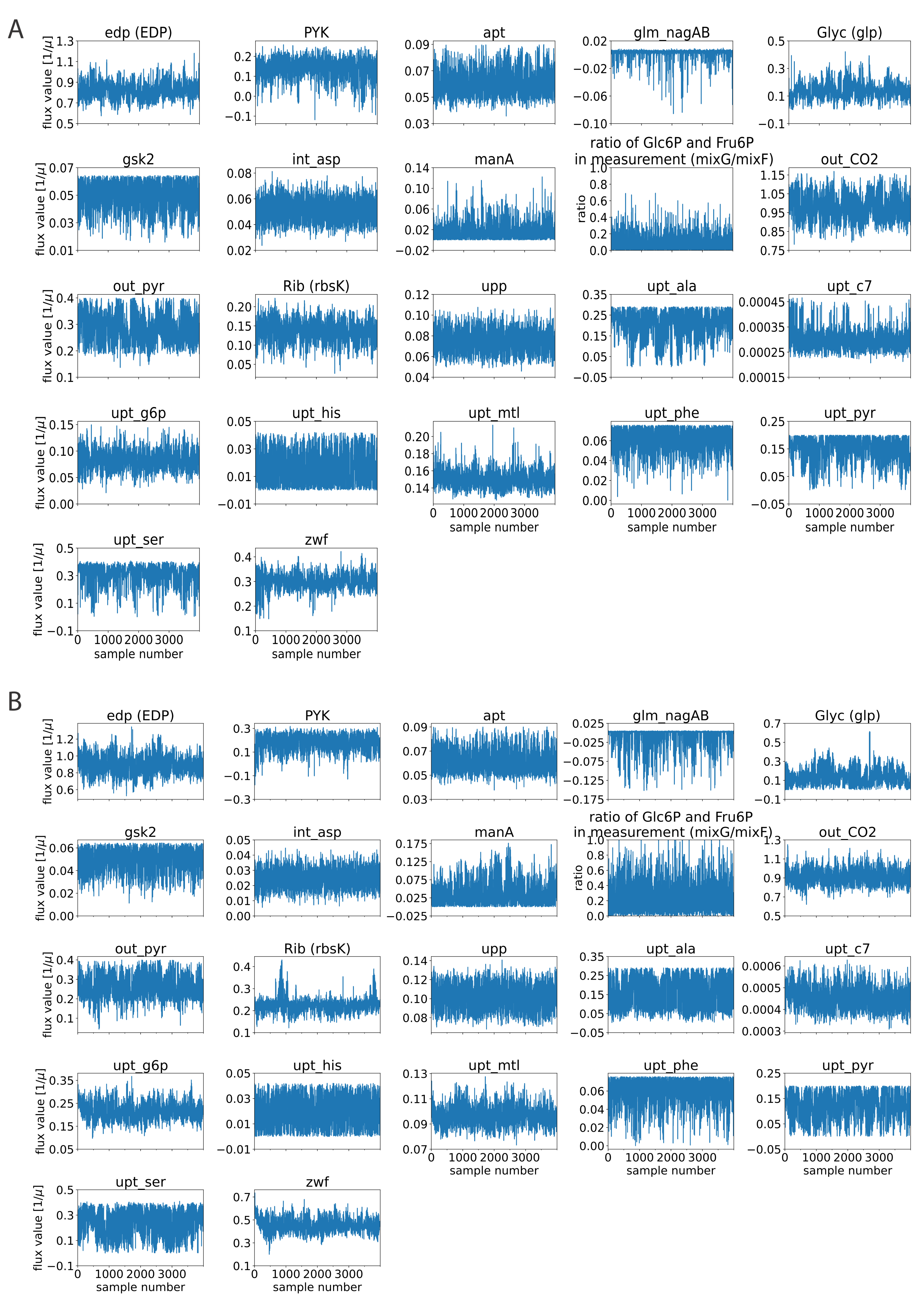

Supplement: S8 Fig — The MCMC algorithm is run for 1.6·107 iterations using 10 independent chains. The first 6·106 samples were treated as burn-in and were, therefore, discarded. Of the remaining 107 samples, each 2,500th sample was saved (thinning), yielding 4,000 samples per MCMC replicate. The trace plots indicate the proper mixing of the Markov chain in the flux space. (A) Low glucose concentration, (B) high glucose concentration. (TIF) [file pbio.3002198.s008.tif]

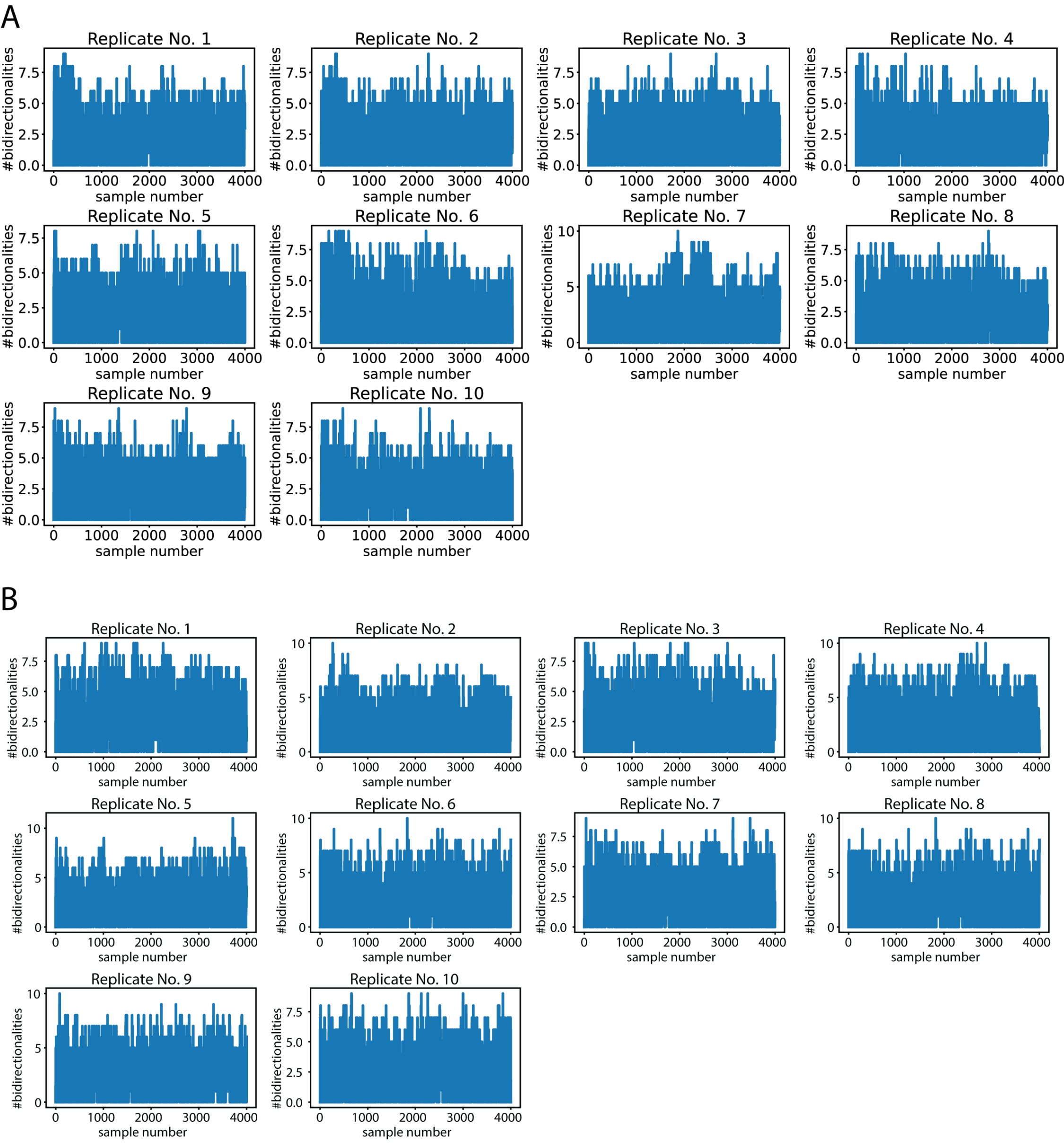

Supplement: S9 Fig — To certify that the replicates did not get stuck in different regions of model space, all replicates are shown. Each subplot represents 1 independent MCMC run. The plots show that the sampler mixes well in the model space and that the mixing is very reproducible for all 10 replicate chains. (A) Low glucose concentration, (B) high glucose concentration. (TIF) [file pbio.3002198.s009.tif]

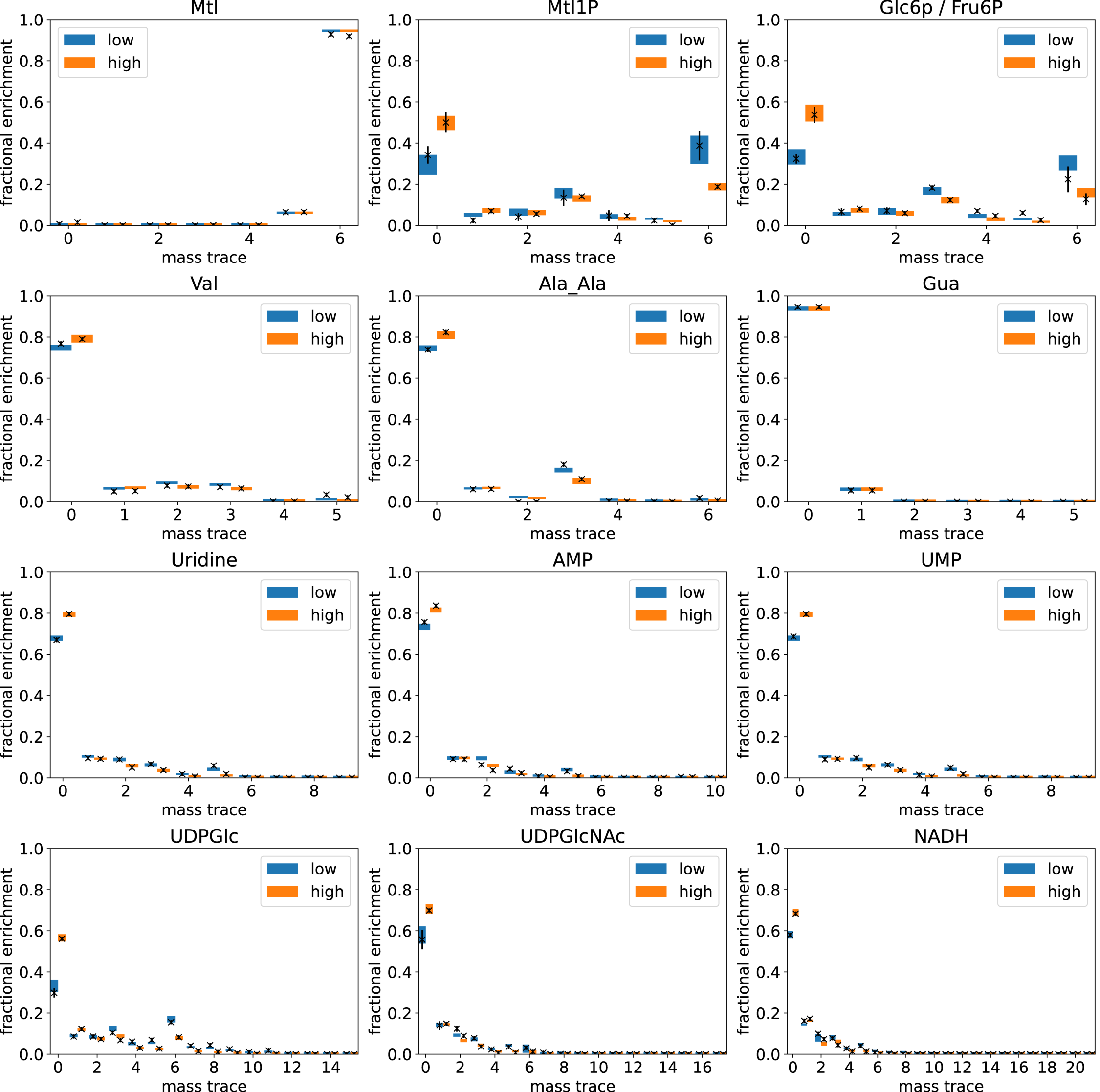

Supplement: S10 Fig — Simulated mass isotopomer distributions (colored bars depict the 95% interval of the simulated measurements), sampled according to the flux posterior distributions obtained from BMA-based 13C MFA inference compared to measured values (black crosses are means with lines as standard deviations). The plots show that the model fits the data well in both cases, the low glucose concentration (blue) and the high glucose concentration (orange). The data underlying this figure can be found in S1 Data. (TIF) [file pbio.3002198.s010.tif]

Repl. 1

Repl. 2

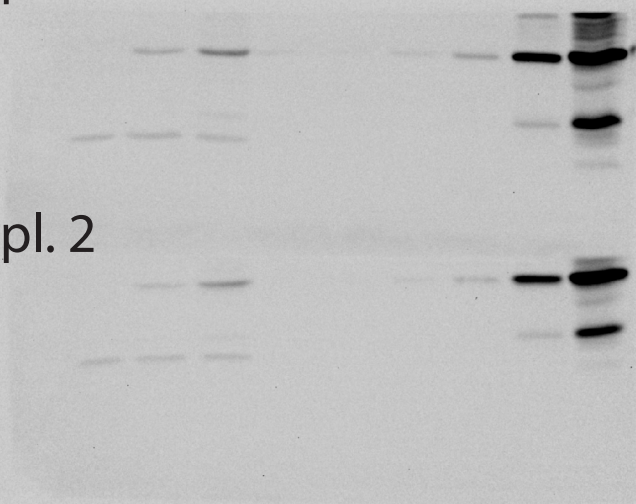

Supplement: S1 Raw Image — The data underlying these images can be found in S1 Data. (PDF) [file pbio.3002198.s011.pdf]
